# Rapid, high-titer biosynthesis of melanin using the marine bacterium *Vibrio natriegens*

**DOI:** 10.3389/fbioe.2023.1239756

**Published:** 2023-09-13

**Authors:** Aaron D. Smith, Tanya Tschirhart, Jaimee Compton, Tiffany M. Hennessa, Eric VanArsdale, Zheng Wang

**Affiliations:** ^1^ United States Naval Research Laboratory, Center for Bio/Molecular Science and Engineering, Washington, DC, United States; ^2^ College of Science, George Mason University, Fairfax, VA, United States; ^3^ American Society for Engineering Education Postdoctoral Research Associate, United States Naval Research Laboratory, Washington, DC, United States; ^4^ National Research Council Postdoctoral Research Associate, United States Naval Research Laboratory, Washington, DC, United States

**Keywords:** *Vibrio natriegens*, melanin, melanin biosynthesis, tyrosinase, tyrosine, biomanufacturing, copper induction

## Abstract

Melanin is one of the most abundant natural biomolecules on Earth. These macromolecular biopolymers display several unique physical and chemical properties and have garnered interest as biomaterials for various commercial and industrial applications. To this end, extensive research has gone into refining methods for the synthesis and extraction of melanin from natural and recombinant sources. In this study, we developed and refined a procedure using a recombinant microbial system for the biosynthesis of melanin using the tyrosinase enzyme Tyr1 and tyrosine as a substrate. Using the emergent microbial chassis organisms *Vibrio natriegens*, we achieved maximal yields of 7.57 g/L, and one of the highest reported volumetric productivities of 473 mg L^−1^ h^−1^ with 100% conversion rates in an optimized, minimally defined medium. Additionally, we identified and investigated the use of a native copper responsive promoter in *V. natriegens* for stringent regulation of heterologous protein expression as a cost effective alternative to traditional IPTG-based induction. This research represents a promising advancement towards a green, rapid, and economical alternative for the biomanufacture of melanin.

## 1 Introduction

Melanin is one of the most abundant biomolecules in the natural environment. Produced by all domains of life, these natural pigments facilitate a vast array of diverse biological functions, demonstrating unique physicochemical properties. Primarily, melanin is associated with organismal or host cell protection, demonstrating a strong ability to absorb a wide spectrum of electromagnetic radiation such as UV light, acting as a strong antioxidant due to its ability to rapidly quench reactive oxygen species (ROS), and providing a strong physical and chemical barrier to the external environment of melanized cells (d'Ischia et al., 2015). Additional unique biological functions have also been reported in specific species or cell types, such as thermoregulation (Cordero et al., 2018), ionizing radiation resistance (Dadachova and Casadevall, 2008), pathogen virulence (Nosanchuk and Casadevall, 2006), antibacterial activity (Correa et al., 2017), and heavy metal chelation (Sarna et al., 2022), to name just a few.

These diverse properties, along with its natural biocompatibility and high biostability, have garnered significant interest from the biomedical and biotechnology fields for the development of novel melanin biomaterials spanning a broad range of applications (Liu R. et al., 2022; Lorquin et al., 2022). However, a significant barrier towards the realization of commercial melanin biomaterials is generating sufficient quantities of melanin in a timely and economical manner. Currently, commercial melanin is either chemically synthesized or extracted from natural sources, such as Sepia ink (Prados-Rosales et al., 2015; Srisuk et al., 2016). These processes have several disadvantages including low availability and product yields, high costs, laborious extraction procedures often requiring harsh chemical treatments, and the generation of hazardous waste streams. This has limited the scope of melanin products to small scale experimental and niche applications thus far.

Biotechnical approaches for whole-cell catalysis using microbes offers a promising alternative for the biomanufacture of melanin. Microbial fermentation offers several advantages including decreased costs, convenient and flexible operating conditions, high catalytic efficiencies, scalability, and most importantly, reactions occur under physiological conditions in aqueous solutions. Several species of bacteria and fungi naturally produce melanin, and cultivation of these species has been explored as an avenue for the bioproduction of melanin. In some instances, metabolic engineering efforts to enhance and/or direct substrate flux for the *de novo* biosynthesis of melanin from carbon sources, such as glucose, have led to improvements in production yields in these natural producers. Alternatively, genes encoding the enzymes responsible for the biocatalysis of melanin have been cloned and heterologously expressed in traditional industrial workhorse strains, such as *E. coli*, for the bioproduction of melanin. To date, production yields and efficiencies of microbial synthesized melanin have steadily improved, but vary wildly depending on several factors including host strain, selection/source of enzyme, method of induction, growth conditions, and the melanin precursor substrate utilized (Martinez et al., 2019; Choi, 2021).

Achieving maximal industrial performance for microbial biomanufacturing processes is highly dependent on two key factors: growth rates and biomass specific substrate uptake rates (Hoffart et al., 2017). The marine bacterium *Vibrio natriegens* has one of the fastest reported doubling times (<10 min) in nutrient rich medium (Payne et al., 1961; Eagon, 1962) and is reported to be over twice as fast as *E. coli* in a defined minimal glucose medium (Long et al., 2017). Additionally, it can utilize a wide variety of carbon sources, demonstrating substrate uptake rates greater than 2-fold compared to *E. coli* (Long et al., 2017; Ellis et al., 2019). As such, *V. natriegens* has become a promising emergent microbial powerhouse for industrial bioprocesses, with potential to dethrone *E. coli* as the gold standard (Lee et al., 2016; Weinstock et al., 2016). Several additional characteristics make *V. natriegens* an attractive industrial bioproduction chassis. Its genome has been fully sequenced and the organism has been phenotypically characterized, demonstrating a very similar core metabolic network compared to *E. coli* (Maida et al., 2013; Wang et al., 2013). Generally, it is considered a safe organism (BSL-1) with no known or reported cases of human infection. It has a significantly higher ribosome content (115,000/cell) than *E. coli* (70,000/cell) (Aiyar et al., 2002), making it a superior recombinant protein expression system or for use in cell-free expression systems (Des Soye et al., 2018; Failmezger et al., 2018; Wiegand et al., 2018; Kormanova et al., 2020). Lastly, *V. natriegens* is a halophile, requiring elevated concentrations of Na^+^ ions for optimal growth, and its high salt tolerance can be exploited for axenic fermentation to circumvent a need for costly antibiotics and prior media sterilization. The surge of interest in using *V. natriegens* as an industrial production strain has led to the recent development of several molecular tools for genetic manipulation and transformations in this organism (Lee et al., 2019; Tschirhart et al., 2019; Stukenberg et al., 2021) as well as genetic engineering efforts that have led to the production of the commercially available Vmax strain (Telesis Bio) (Becker et al., 2019) making this organism accessible and easy to genetically manipulate in essentially any molecular biology laboratory.

There have been several recent examples in the literature using *V. natriegens* as a platform for the biosynthesis of various relevant value-added chemicals through recombinant protein expression, metabolic engineering, strain engineering, or a combination of these methods. These include the biosynthesis of various amino acids (Stella et al., 2021), succinate (Thoma et al., 2022), polyhydroxybutyrate (Chien et al., 2007), 2,3-butanediol (Meng et al., 2022), β-carotene and violacein (Ellis et al., 2019), 1,3-propanediol and 3-hydroxypropionate (Zhang et al., 2021), and L-DOPA (Liu X. et al., 2022), to name just a few. In particular, the high-efficiency production of L-DOPA, a key melanin precursor, hints at the promising capability of biosynthesizing high yields of melanin in this organism. Previously, our lab investigated the ability of *V. natriegens* to biosynthesize melanin when heterologously expressing the tyrosinase enzyme Tyr1. This resulted in the successful biocatalysis of tyrosine to melanin which was subsequently characterized. However, while yields were low, melanin production was complete within 10 h compared to *E. coli* which required more than 24 h (Wang et al., 2020), a significant time and cost savings when considering the economics of scale-up manufacturing.

While significant strides have been made to improve yields and production efficiencies, there still remain significant barriers to large scale, cost efficient production of melanin. In this study, we performed a systematic comparison of different classes of melanin-producing enzymes in both the model bacterium *E. coli* and the emergent microbial chassis organism *V. natriegens* for the biosynthesis of melanin, with an emphasis on optimizing maximal product yields and production efficiencies. We also assessed the ability to induce protein expression using various promoter systems to develop a strain capable of rapid, high yield melanin production. Further, we developed and optimized defined minimal media for high density growth of *V. natriegens* to facilitate melanin production in both cultures flasks and bioreactors. In total, from overnight cultures of *V. natriegens* we were able to biosynthesize melanin at a maximal capacity of 7.57 g/L representing a volumetric productivity of 473 mg L^−1^ h^−1^, one of the highest reported in the literature to date. Lastly, we also developed a modified medium for transitioning high capacity melanin production into bioreactors, demonstrating potential for future scale-up endeavors and providing a promising route towards the economical production of melanin biomaterials.

## 2 Materials and methods

### 2.1 Materials, strains, and growth conditions

All chemicals and reagents were purchased from Sigma Aldrich unless otherwise specified. Cloning and plasmid propagation were conducted in *E. coli* NEB10β cells (New England Biolabs) according to manufacturer specifications. Recombinant protein expression studies and melanin production were conducted in *E. coli* BL21 (DE3) cells (New England Biolabs). *E. coli* was grown in LB (Luria broth [Miller]) or M9 media (1X M9 salts (BD Biosciences), 1 mM MgCl_2_, 0.1 mM CaCl_2_, 0.2% (w/v) glucose) unless otherwise stated and supplemented with ampicillin (100 μg/ml) or chloramphenicol (25 μg/ml) as required for plasmid maintenance. All studies conducted with *V. natriegens* were performed with strain ATCC 14048. *V. natriegens* was grown in LBv2 (Luria broth [Miller] supplemented with 204 mM NaCl, 4.2 mM KCl, and 23.14 mM MgCl_2_) or VnM9v2 (see formulation below) media as indicated. For bioreactor studies, *V. natriegens* was grown in VnM9v3 medium (see formulation below). *V. natriegens* cultures were supplemented with chloramphenicol (25 μg/ml) for plasmid maintenance.

### 2.2 Plasmid construction

List of all oligonucleotide primers and sequences used for plasmid construction are listed in Supplementary Table S1. List of all plasmids used in the following studies are listed in Supplementary Table S2. Synthesis of oligonucleotide primers and sequencing verification of plasmids were conducted by Eurofins Genomics LLC.

#### 2.2.1 IPTG-inducible plasmids

The inducible pJV-Tyr1 plasmid containing *Priestia megaterium* (formerly *Bacillus megaterium*) tyrosinase (Tyr1) used in this study was previously published by our group (Wang et al., 2020). The sequence of *Rhizobium etli melA* gene was obtained from the NCBI database and synthesized by Eurofins. The *E. coli hpaBC* gene sequence in the plasmid pRSF1010 was kindly provided by Dr. Nathan Schwalm at Army Research Laboratory. The *hpaBC* genes are expressed in tandem from a single operon. Using Q5 High Fidelity Polymerase (New England Biolabs), the *melA* and *hpaBC* gene sequences were amplified with primer pairs MelA-pJ_F/MelA-pJ_R and HpaBC-pJ_F/HpaBC-pJ_R, respectively. The pJV298 vector backbone was amplified with primer pairs pJV_melA_F/pJV_melA_R and pJV_hpaBC_F/pJV_hpaBC_R for MelA and HpaBC, respectively. The genes for *melA* and *hpaBC* were cloned into the pJV298 plasmid backbone in a similar manner as was done previously for *tyr1* using the HiFi Gibson Assembly Master Mix (New England Biolabs) (Wang et al., 2020).

#### 2.2.2 Constitutive plasmids

The pCB1D5-GFP plasmid (DVC) (Tschirhart et al., 2019) was used as the backbone for initial cloning of the *tyr1*, *hpaBC*, and *melA* genes. The pCB1D5 plasmids contain the constitutive Anderson promoter J23106 and RBS B0032 upstream of the cloned genes, and was constructed using the HiFi Gibson Assembly Master Mix (New England Biolabs) (iGEM, 2023). The vector was amplified using Q5 High Fidelity Polymerase (New England Biolabs) with the primer pairs pCB1D5_tyr_F/pCB1D5_tyr_R, pCB1D5_hpaBC_F/pCB1D5_hpaBC_R, and pCB1D5_melA_F/pCB1D5_melA_R for Tyr1, HpaBC, and MelA assembly, respectively. The *tyr1*, *hpaBC*, and *melA* genes were amplified using primer pairs Tyr1-pC_F/Tyr1-pC_R, HpaBC-pC_F/HpaBC-pC_R, and MelA-pC_F/MelA-pC_R, respectively. The Gibson DNA assembly reaction was set up and run as indicated by the manufacturer’s protocol, and 2 µL was transformed into NEB10β chemically competent cells as per manufacturer’s instructions.

#### 2.2.3 Copper-inducible plasmid

The copper inducible pCopA-Tyr1 plasmid was generated by cloning a 205 bp fragment upstream from the start of the open reading frame of the *V. natriegens copA* gene into the pCB1D5 plasmid backbone using the In-Fusion HD cloning kit (Takara Bio) per manufacturer instructions. Primers pCopA_F and pCopA_R, having complementary overhangs to the pCB1D5-Tyr1 plasmid, were used to amplify this region by PCR using Q5 High Fidelity Polymerase (New England Biolabs). The pCB1D5 backbone was amplified from the pCB1D5-Tyr1 plasmid using the primer sets pCB1D5_copA_F and pCB1D5_copA_R in a similar manner, specifically omitting the promoter regions and the *tyr1* gene. Tyr1 was re-amplified using primers Tyr1_copA_F, containing complementary overhangs with the *copA* promoter fragment and Tyr1_copA_R, containing complementary overhangs with the pCB1D5 plasmid backbone.

### 2.3 *V. natriegens* electrocompetent cell preparation and transformation

Electrocompetent cells were prepared as previously described. (Tschirhart et al., 2019). Aliquots (50 μL) of competent cells were thawed on ice and mixed gently with plasmid DNA (100 ng total). The cell–DNA suspension was transferred to a chilled electroporation cuvette with a 0.1 cm gap size. Cells were electroporated using the following parameters: 900 V, 25 μF, 200 Ω. The cells were then immediately recovered in 500 μL of recovery medium (BHIv2 + 680 mM sucrose) and transferred to a 15 ml conical tube and incubated at 37 °C for 1.5 h. Aliquots of the recovery medium were plated out on warm agar plates containing the appropriate antibiotic and incubated for several hours or overnight at 37 °C.

### 2.4 Screening melanin production in *E. coli*

*E. coli* BL21 (DE3) cells were used for the initial screens of enzyme expression and melanin production. Cells transformed with the indicated plasmids were inoculated into 50 ml of M9 or LB media as indicated from overnight starter cultures to a starting OD_600_ of 0.1. Cells were incubated at 37 °C, 200 rpm until reaching an OD_600_ ∼0.8. At this point, cultures were induced with 1 mM isopropyl β-d-1-thiogalactopyranoside (IPTG) (only for pJV plasmids) and supplemented with 2 g/L tyrosine and 40 µM CuSO_4_ (only for Tyr1 and MelA expressing cultures). Control cultures were grown in parallel that were not supplemented with tyrosine. Cultures continued to incubate at 200 rpm 37 °C for up to 24 h. MelA expressing cultures continued incubation for up to 6 days 1 ml aliquots were collected at the indicated timepoints for OD_600_ and A_492_ readings and for protein expression analysis. OD_600_ values were recorded from control cultures not synthesizing melanin. A_492_ values were recorded from cell-free culture supernatants.

### 2.5 Minimal media optimization

Optimization of a minimally defined medium supporting high density *V. natriegens* growth was performed using a Bioscreen C 96-well microtiter plate assay. Initially, a base M9 medium was used as a basis for growth evaluation, with the addition of extra sodium chloride required to support *V. natriegens* growth. The initial *V. natriegens* base M9 medium consisted of 50 mM sodium phosphate (pH 7.4), 20 mM NH_4_Cl, 1 mM Na_2_SO_4_, 1 mM MgCl_2_, 0.1 mM CaCl_2_, 200 mM NaCl, and 20 mM glucose. From the base medium, one component was varied in concentration while all others were kept constant. Cultures were inoculated to a starting OD_600_ = 0.05 from an overnight culture and 200 µL added to 100 well honeycomb microtiter plates. After establishing an optimized base medium, additional components such as amino acids, vitamins, minerals, and metals were sequentially added and varied across a set of concentration ranges to test for growth enhancement. Ultimately, conditions supporting rapid, high density growth of *V. natriegens* was determined: 80 mM K_2_HPO_4_ phosphate, 20 mM NaH_2_PO_4_ (pH 7.4), 50 mM NH_4_Cl, 5 mM Na_2_SO_4_, 275 mM NaCl, 1 mM MgSO_4_, 0.3 mM CaCl_2_, 20 mM glucose, 0.4% (w/v) glycerol, 0.2% (w/v) casamino acids, 0.2% (w/v) aspartate, and 0.2X trace metals mix. A stock of 1,000X trace metals was used to supplement cultures consisting of: 50 mM FeCl_3_, 10 mM MnCl_2_, 10 mM ZnSO_4_, 2 mM CoCl_2_, 2 mM CuCl_2_, 2 mM NiCl_2_, 2 mM Na_2_MoO_4_, 2 mM Na_2_SeO_3_, and 2 mM H_3_BO_3_. All conditions screened were conducted in biological triplicates.

### 2.6 Protein expression analysis

Recombinant enzyme production in *E. coli* or *V. natriegens* was induced with 1 mM IPTG. 1 ml aliquots were collected pre-induction and at various time points post-induction, as indicated. Cells were pelleted 10,000 x g for 2 min, culture supernatants decanted, and cell pellets stored at -80 °C until ready for analysis.

Cell extracts were prepared by resuspending cell pellets in lysis buffer consisting of 50 mM Tris (pH 7.5), 50 mM NaCl, 2 mM MgCl_2_, 2 mM EDTA, 0.1 mg/ml lysozyme, 1 U/ml DNase I, and 1 mM PMSF. Cells were incubated at room temperature for 20 min, then a stock solution of 10% (w/v) SDS was added to cultures to a final concentration of 0.1% (w/v). Cells were placed on ice and pulse sonicated with a microtip sonicator (Qsonica) for 1s on, 1s off, for 10s total at 25% amplitude. Lysed cells were centrifuged at 16,000 x g for 10 min and soluble cell extracts collected. Total protein concentrations were determined by BCA assay (Roche) and samples were mixed with a 4X SDS-loading buffer (BioRad) with added 2-mercaptoethanol and heated at 90 °C for 5 min 20 µg of total protein were loaded onto 4%–15% SDS-PAGE gels (BioRad) for separation of proteins and visualized by staining with GelCode Blue (Thermo Fisher Scientific).

### 2.7 Melanin biosynthesis in *V. natriegens*

*V. natriegens* cells harboring the indicated plasmids were incubated in 5 ml VnM9v2 medium overnight at 37 °C, 200 rpm. The following day, 1/100 volume of culture was used to inoculate fresh VnM9v2 medium and incubated at 37 °C, 200 rpm, until the OD_600_ reached ∼0.8. Protein expression was induced with 1 mM IPTG for cells harboring the pJV plasmid or 100 µM CuSO_4_ for cells harboring the pCopA plasmid. The cultures were incubated for an additional 3 h, 200 rpm, at 37°C or 30°C as indicated. After the induction period, melanin production was initiated by supplementing cultures with 40 µM CuSO_4_ (for pJV-Tyr1) and the indicated concentrations of tyrosine or disodium tyrosine. Cultures were incubated at 37 °C or 30 °C as indicated, 200 rpm, overnight.

### 2.8 Melanin purification

Cultures were pelleted at 6,000 x g for 10 min, and melanin-containing supernatant was decanted to a separate flask. Melanin contained within the supernatant (“supernatant melanin”) was precipitated by addition of 6 M HCl until the pH dropped to <2. Precipitated melanin was centrifuged 10,000 x g for 10 min. Acidified supernatant was discarded and pelleted melanin was solubilized in 1 M NaOH and incubated in a water bath at 80 °C for 1 h. The samples were cooled to room temperature and again precipitated by addition of 6 M HCl to pH < 2 and incubated at room temperature for 30 min. This acid/base cycling procedure was repeated for a minimum of three cycles.

Pelleted cultures containing intracellular melanin and/or cellular associated melanin (“biomass melanin”) were resuspended in 1 M NaOH and incubated in a water bath at 80 °C for 1 h. Samples were cooled to room temperature and then briefly sonicated on ice for 3 min at 60% duty cycle and output setting of 6 using a Branson Sonifier 450 sonicator. The melanin was precipitated by addition of 6 M HCl until the pH was <2 and centrifuged at 10,000 x g for 10 min. This acid/base cycling procedure was repeated for a minimum of three cycles as above for the supernatant melanin. After acid/base washing, HCl precipitated melanin was washed 3x in water. Melanin pellets were placed in a 65 °C oven until dried (24–48 h).

### 2.9 Melanin toxicity assays

CFU/mL measurements of melanin toxicity were determined by first growing either *V*. *natriegens* or *E*. *coli* BL21 (DE3) cells to mid-log phase (OD_600_ ∼0.5–0.7) then re-inoculating the cells at a 1:100 ratio into 1 ml of M9 or VnM9v2 media with a concentration of melanin between 0 and 10 g/L. Purified melanin was pre-incubated in the media for 3 h (37 °C, 220 rpm) prior to adding to the re-inoculation media. Samples were then grown for 2 h at 37 °C, 220 rpm, then serially diluted in PBS for *E. coli* or PBS with 300 mM NaCl for *V. natriegens.* 5 μL of each suspension was then spotted onto LM or LB plates respectively and grown overnight in a 37 °C incubator. CFU/mL measurements were calculated at the greatest dilution factor that had at colonies for least three biological replicates.

### 2.10 *V. natriegens* bioreactor growth and melanin biosynthesis

A starter culture was used to inoculate 2 × 100 ml of VnM9v3 in a DASGIP bioreactor (Eppendorf) to a starting OD_600_ of 0.1. Bioreactor process parameters were set for a temperature of 30 °C, pH set point of 7.0, and a dissolved oxygen (DO%) setpoint of 30%. DO% was controlled by an automated cascade with the following settings: N: 0%–40%, 400–1,200 rpm; XO_2_: 40%–80%, 21% constant; and F: 40%–100%, 6–18 s L/h. pH was adjusted by automatic injections of acid or base from the DASGIP pump controller using 3 M NaOH or 1 M H_3_PO_4_ stocks. A foam level detector was also used, with automated antifoam injections of a 1% PPG2000 controlled by a custom script set up in the DASGIP control software. OD_600_, A_492_, and pH values were monitored throughout the run. 1 mM IPTG, 40 µM CuSO_4_, and 8 g/L disodium tyrosine were injected through injection ports on the bioreactor vessels for Tyr1 induction and initiating melanin biosynthesis. At the end of the run, cultures were collected and melanin was purified as described above using the acid/base cycling procedure to obtain final yields.

### 2.11 mRNA isolation and qRT-PCR analysis of transcript levels

#### 2.11.1 mRNA purification

*V. natriegens* overnight cultures grown in VnM9v2 medium were diluted to OD_600_ of 0.2 in 5 ml fresh VnM9v2, and recovered at 37 °C, 200 rpm until OD_600_ values reached 0.6–0.8. At this point, cultures were supplemented as indicated to induce gene expression. Cultures were incubated further for 3 h at 30 °C, then centrifuged 5,000 x g for 10 min to collect cells for mRNA isolation using the Total RNA Isolation Kit (New England Biolabs) following manufacturer recommendations. mRNA was eluted in 90 µL nuclease-free water. 10 µL of 10X DNase Reaction Buffer and 5 µL DNase I (Epicentre) was added to purified mRNA and incubated at 37 °C for 1 h. Samples were diluted with 100 µL nuclease-free water and mRNA purified by acid phenol:chloroform extraction and ethanol precipitation using standard protocols (Sambrook and Russell, 2006). Purified mRNA was resuspended in 50 µL RNase-free water and concentrations were determined using a Nanodrop One C (Thermo Fisher Scientific).

#### 2.11.2 cDNA synthesis

mRNA samples were adjusted to 100 ng/μL with nuclease-free water. 10 µL purified RNA (1 µg total) was used to synthesize cDNA using the iScript cDNA synthesis kit (BioRad) in a 20 µL total reaction volume according to manufacturer recommendations. Control reactions containing no reverse transcriptase were included. After cDNA synthesis, reactions were diluted by adding 180 µL nuclease-free water.

#### 2.11.3 qRT-PCR

qRT-PCR analysis of transcript levels were conducted using a SYBR Green Master Mix (BioRad) according to manufacturer recommendations in a 10 µL total reaction volume. Reactions were analyzed using a Step One ThermoCycler (Applied Biosystems), and C_T_ values were determined using the included software. Target gene expression values were normalized to the housekeeping gene *gapA* and expression fold changes determined by the ΔΔC_T_ method. All primers for qRT-PCR studies are listed in Supplementary Table S1. For all qRT-PCR studies, technical duplicates of independent biological triplicates were used for the analysis of mRNA expression profiles.

## 3 Results

### 3.1 Recombinant protein expression and melanin biosynthesis in *E. coli*

**FIGURE 1:**
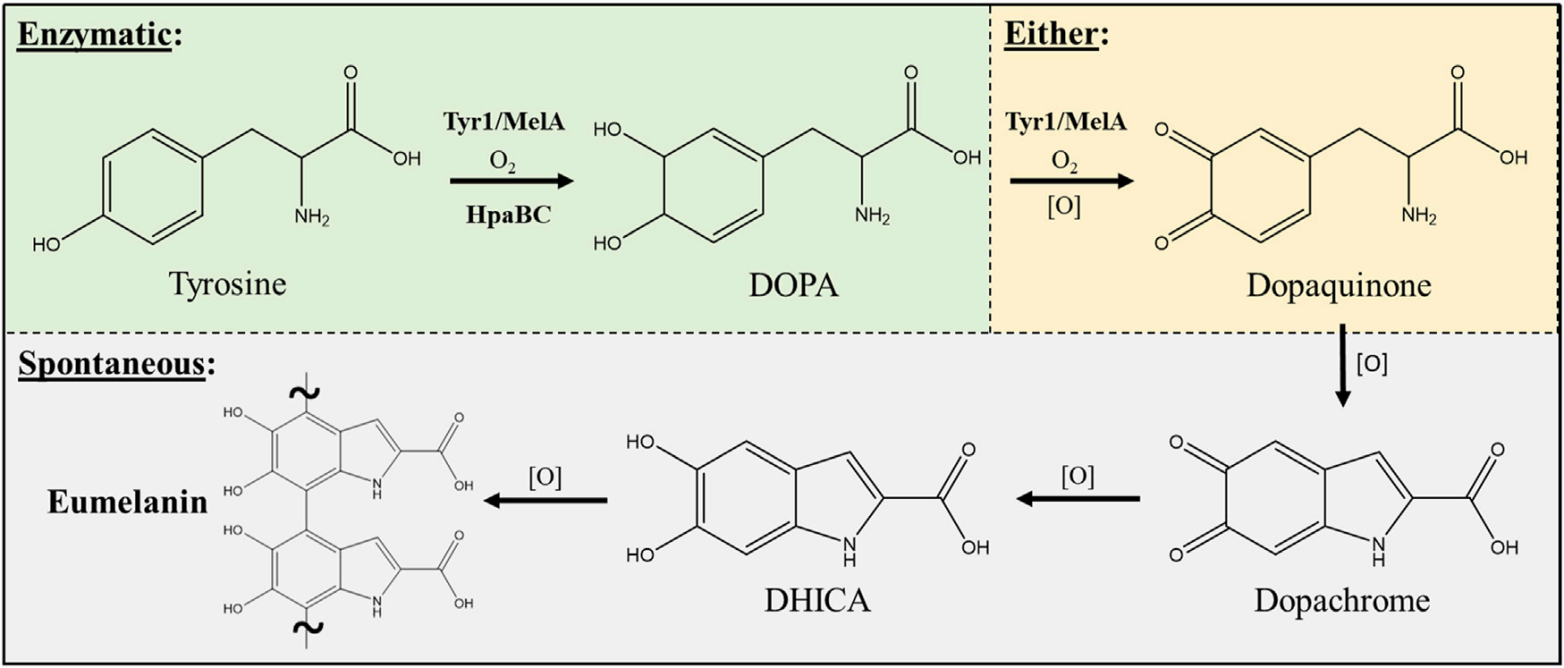
Schematic portraying the enzymatic and spontaneous reactions for biosynthesis of eumelanin starting from L-tyrosine as a substrate and intermediates along the biosynthetic pathway. The conversion of DOPA to dopaquinone can be either enzymatic via a tyrosinase (i.e. Tyr1 or MelA) or can occur spontaneously in the presence of oxidants [O] such as molecular oxygen. All enzymatic steps along the pathway require molecular oxygen (O_2_) for the initial oxidation reactions. DOPA–3,4-dihydroxyphenylalanine; DHICA–5,6-dihydroxyindole-2-carboxylic acid.

Three enzymes were initially selected to screen for melanin production efficiencies when heterologously expressed in *E. coli* (Table 1). These included the tyrosinases Tyr1 from *Priestia megaterium* (Shuster and Fishman, 2009) (formerly *Bacillus megaterium* (Gupta et al., 2020)) and MelA from *Rhizobium etli* (Cabrera-Valladares et al., 2006). Tyrosinase (EC# 1.14.18.1) is a classic melanizing enzyme, utilizing a catalytic redox-active dinuclear copper center for subsequent enzymatic oxidations of tyrosine to L-DOPA, and L-DOPA to dopaquinone, which then spontaneously auto-oxidizes and polymerizes to melanin (Figure 1). A third, separate class of enzyme consisting of a two-component 4-hydroxyphenylacetate 3-monooxygenase (HpaB) and its associated NADH-dependent reductase (HpaC) from *E. coli* was also evaluated for its ability produce melanin (Prieto et al., 1993; Fordjour et al., 2019). HpaB (EC# 1.14.14.9) utilizes FADH_2_ as a cofactor and can oxidize a variety of mono-phenolic substrates. Oxidized FAD in HpaB is recycled by equivalents of NADH supplied by the reductase HpaC (EC# 1.5.1.37). In contrast to tyrosinase, HpaB is only capable of performing the single oxidation of tyrosine to L-DOPA, a key precursor in melanin biosynthesis, which can then auto-oxidize non-enzymatically to melanin in the presence of oxidants (Figure 1). Previous studies using HpaBC to biosynthesize L-DOPA typically supplement with a reducing agent, such as ascorbate, in the medium to prevent auto-oxidation to melanin (Fordjour et al., 2019).

**TABLE 1:** List of enzymes tested in this study for the bioproduction of melanin and some of their characteristic features. HpaB and HpaC are co-expressed together. EC#–Enzyme commission number. MW–Molecular weight.

Enzyme	Species	EC#	Required cofactor	MW (kDa)
HpaB	*Eschericia coli*	1.14.14.9	FADH2	59.0
HpaC	*Eschericia coli*	1.5.1.37	NADH	18.5
MelA	*Rhizobium etli*	1.14.18.1	Cu (2 mol eq.)	55.3
Tyr1	*Priestia megaterium*	1.14.18.1	Cu (2 mol eq.)	34.4

**FIGURE 2:**
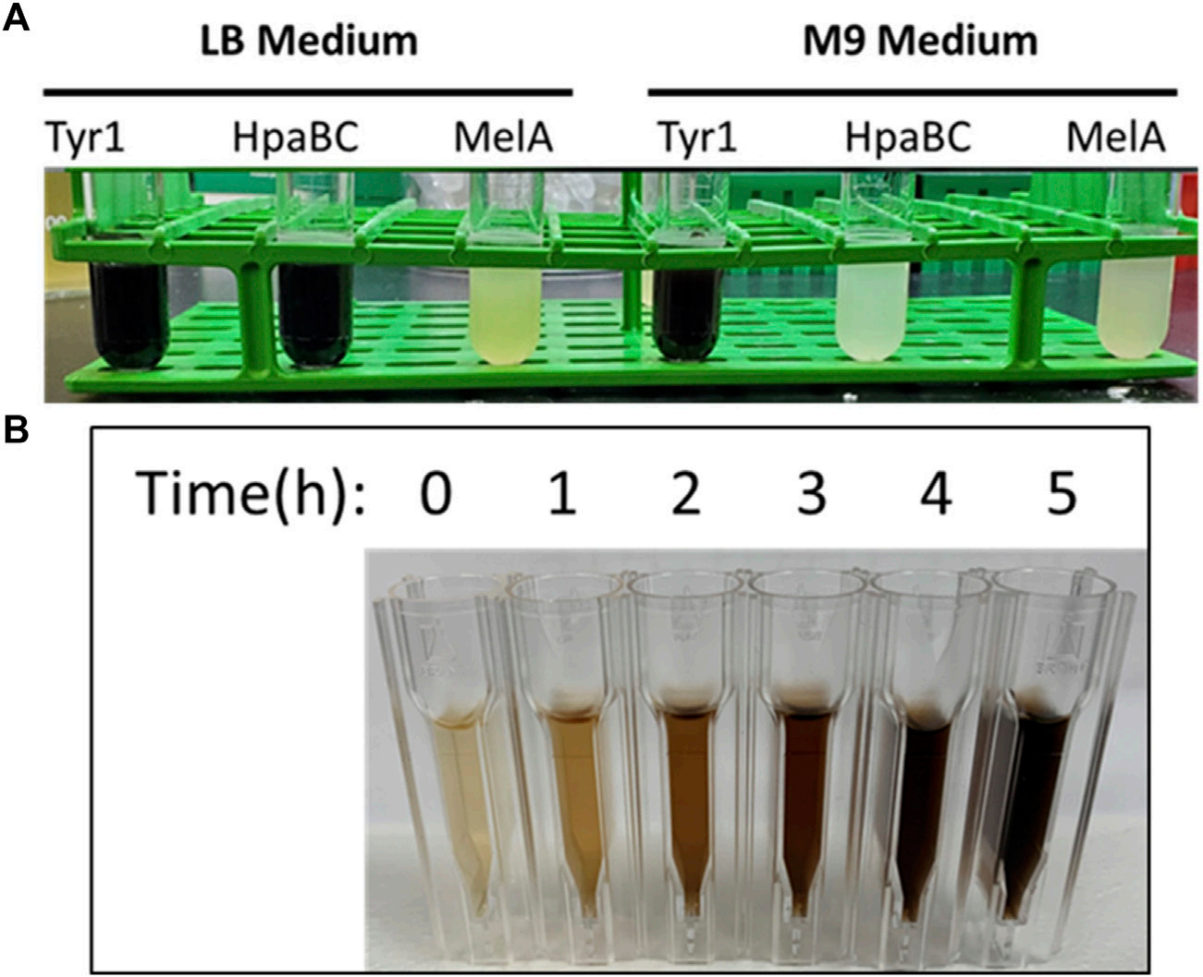
**(A)** Image of melanin producing *E. coli* BL21 (DE3) cells transformed with the IPTG-inducible pJV plasmid expressing the indicated enzymes. Cultures were induced with 1 mM IPTG, supplemented with 2 mg/ml tyrosine, and allowed to incubate for 24 h at 37 °C. The visible dark, black pigmentation is characteristic of melanin formation. **(B)** Visualization of melanin formation in cell-free culture supernatants from pJV-Tyr1 expressing *E. coli* BL21 (DE3) cells. Melanin formation was monitored spectrophotometrically by recording absorbance values at 492 nm. Time points above indicate time post-induction with IPTG and addition of tyrosine.

The genes for *tyr1*, *melA*, and *hpaBC* were cloned identically into either the pJV or pCB1D5 plasmids. In the pJV plasmid, gene expression is controlled by the *Ptac* promoter and *LacI* ^*Q*^ repressor for inducible control of gene expression by IPTG (van Kessel et al., 2015). Conversely, the pCB1D5 plasmid maintains constitutive control of gene expression using the *J23106* promoter and the B0032 RBS identified from the Anderson collection (iGEM, 2023). These plasmids were transformed into *E. coli* BL21 (DE3) cells for initial screening and comparative analysis of protein production and melanin biosynthesis when grown in nutrient rich LB or standard M9 minimal media. As seen in Figure 2A, initial screening of overnight *E. coli* cultures expressing the indicated enzymes led to varying degrees of melanin production as evidenced by the formation of the characteristic dark brown/black pigment. Further, melanin formation and enzyme activity appeared to be media specific for the different enzymes tested. To assess the dynamics of melanin production using the various enzyme and promoter systems described above, we performed a spectroscopic time course analysis of melanin formation in culture-free supernatants. As an example, melanin formation began quite rapidly after the addition tyrosine substrate to Tyr1 expressing *E. coli* cells as evidenced by the pigmentation of culture medium over time (Figure 2B). Subsequently, *E. coli* cells expressing HpaBC, Tyr1, or MelA using the different promoter systems and in LB or M9 media were evaluated for cellular growth (OD_600_), melanin formation (A_492_), and protein expression analysis (SDS-PAGE).

**FIGURE 3:**
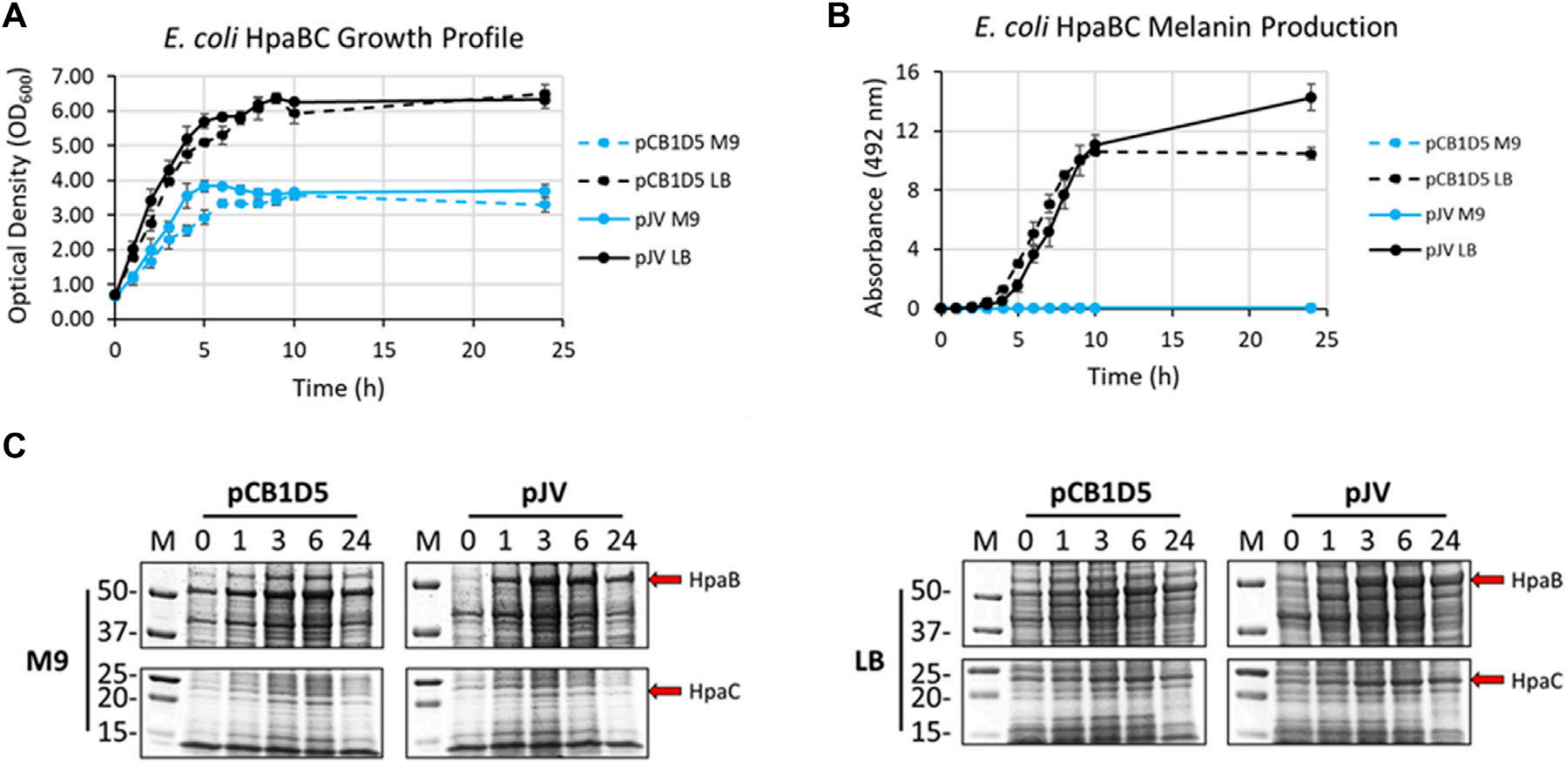
**(A)** Optical density readings (OD_600_) of recombinantly expressed HpaBC in *E. coli*. Cultures were grown in M9 (blue traces) or LB (black traces) media and HpaBC was expressed constitutively from the pCB1D5 plasmid (dashed lines) or induced by 1 mM IPTG using the pJV plasmid (solid lines). Measurements were taken from control cultures not supplemented with tyrosine. **(B)** Absorbance readings at 492 nm (A_492_) for the detection of melanin from recombinantly expressed HpaBC in *E. coli* as in Figure 2A. Cultures were supplemented with 2 mg/ml tyrosine as substrate. A_492_ readings taken on cell-free culture supernatants after centrifugation. Measurements taken from three independent biological replicates. **(C)** HpaBC SDS-PAGE protein expression analysis of *E. coli* soluble cell extracts with the indicated plasmids in either M9 (left) or LB (right) media. For all figures, indicated time points are post-induction with IPTG (for pJV plasmids) and after addition of substrate trysosine. 20 µg total protein loaded in each lane.

Naturally, *E. coli* cells grew better in nutrient rich LB vs. minimal M9 media when expressing HpaBC from either the pJV or pCB1D5 plasmid (Figure 3A). There was no apparent difference in growth between the different plasmids used. However, melanin synthesis was only evident in cultures grown in LB, with no detection of melanin in M9 after 24 h of incubation (Figure 3B). In LB, maximal melanin production began around 5 h and started to saturate after 9 h. However, HpaBC expressed from the pJV plasmid showed a modest increase in melanin production out to 24 h, compared to the pCB1D5 plasmid which did not show an increase in A_492_ values after 10 h. HpaB also demonstrated excellent protein expression in both M9 and LB as evident by an overexpressed band above the 50 kDa marker (predicted MW ∼59 kDa) (Figure 3C). Expression from the pCB1D5 plasmid demonstrated a strong, constitutive expression pattern for HpaB in both media tested, while expression from pJV showed strong induction after the addition of IPTG. It was peculiar that despite strong expression of HpaB in M9, there was no detectable melanin formation. In contrast to HpaB, it appears HpaC (predicted MW ∼15 kDa) is poorly expressed in M9 (Figure 3C), suggesting HpaC may not be expressed or be stable under these conditions and provides a plausible explanation for the lack of melanin formation in M9 medium. Alternatively, the conditions may not be amenable for subsequent auto-oxidation of the enzymatically derived product, L-DOPA, into melanin. We did a quick experiment to test this by supplementing media alone or *E. coli* expressing HpaBC cultures with L-DOPA, instead of tyrosine (Supplementary Figure S1). L-DOPA was auto-oxidized to melanin in media alone as evidenced by the formation of the black melanin pigment. Melanin formation was also apparent with *E. coli* cultured in LB, but not in M9 medium, indicating *E. coli* cell cultures in M9 do not provide an oxidizing enough environment for the auto-oxidation of L-DOPA to melanin.

**FIGURE 4:**
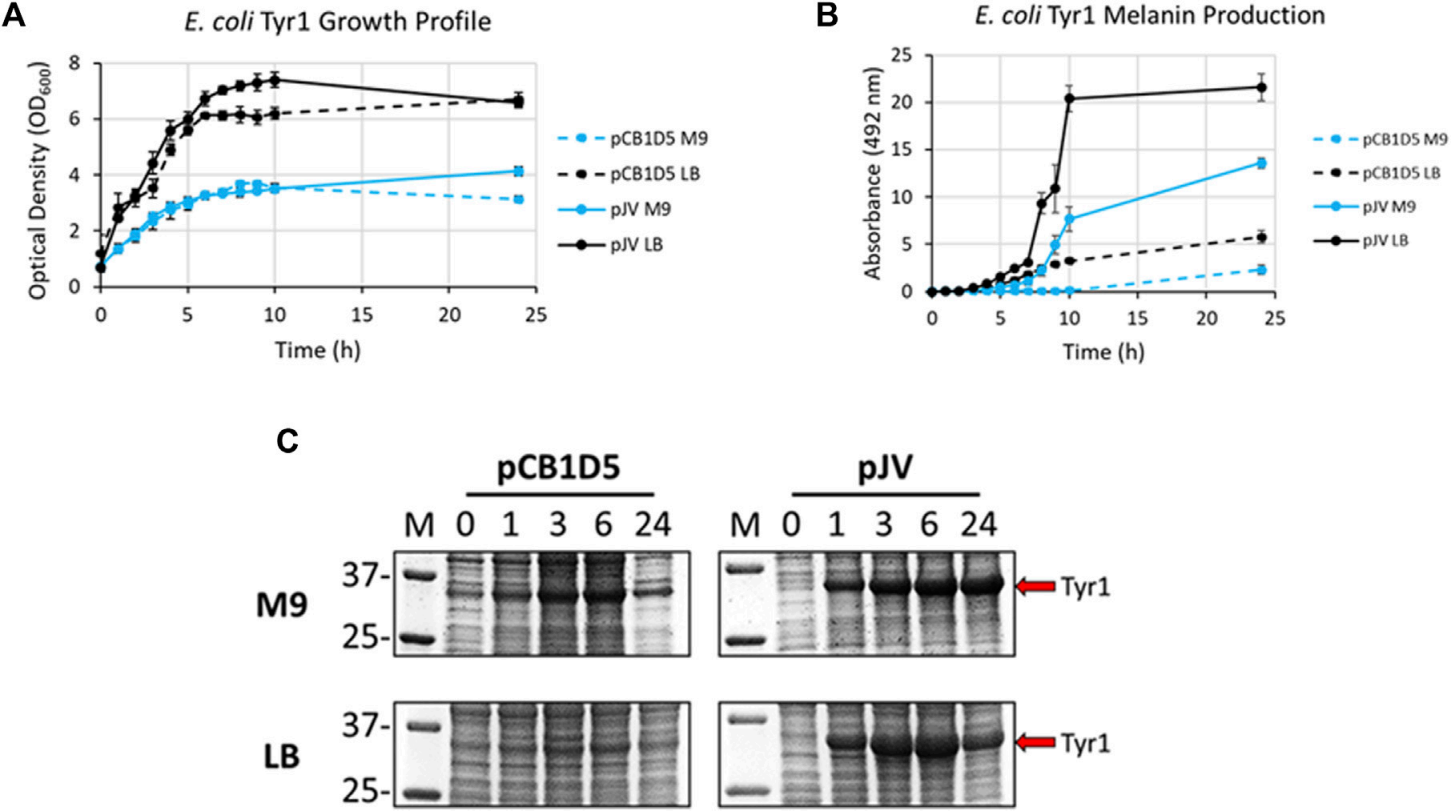
Analysis of growth profile **(A)**, melanin production **(B)**, and protein expression **(C)** as in Figure 3, but for recombinantly expressed Tyr1 in *E. coli* with either the constitutive pCB1D5 or inducible pJV plamsids in M9 or LB medium as indicated.

*E. coli* cells expressing Tyr1 showed a similar growth profile to the HpaBC expression cultures, with better growth in LB vs. M9 media and no apparent differences between the two plasmids used (Figure 4A). In contrast to melanin biosynthesis with HpaBC, melanin formation was detected under all conditions tested for Tyr1, with distinct categorical differences between them (Figure 4B). Melanin synthesis was superior when Tyr1 was expressed from the pJV plasmid vs. the pCB1D5 plasmid. Similarly, between the media tested melanin production was highest in LB vs. M9. However, melanin production appeared to saturate after 10 h in LB, while melanin production continued out to 24 h in M9. Control cultures not supplemented with tyrosine showed no detectable melanin synthesis (data not shown). Protein expression profiles were similar to that seen for HpaB, with an over-expressed protein band near the predicted molecular weight of Tyr1 (predicted MW ∼34 kDa). There was strong, constitutive expression from the pCB1D5 plasmid and a strong, inducible expression of Tyr1 after addition of IPTG (Figure 4C). Interestingly, it did appear after 24 h there was a reduction in Tyr1 levels in LB, while Tyr1 levels remained significantly elevated at this same time point in M9, perhaps explaining the increased production of melanin out to 24 h. Also, it is noteworthy to point out the A_492_ readings for pJV-Tyr1 in M9 medium were comparable to those seen for the maximal production of melanin for HpaBC in LB medium, indicating Try1 performed just as well, if not better, than HpaBC but in a defined minimal medium.

Lastly, melanin production was evaluated using MelA in a similar manner. Growth curves were very similar compared to both HpaBC and Tyr1 (Supplementary Figure S2A). However, there was no visual indication of melanin production under any condition tested within the course of the 24 h experiment. Previous studies expressing MelA in *E. coli* demonstrated a lag in melanin production by nearly 24 h (Cabrera-Valladares et al., 2006). By extending culture incubation periods over the course of a week, melanin formation was observed beginning around day 3, with maximal production achieved by day 4 in the pJV plasmid (Supplementary Figure S2B). In the pCB1D5 plasmid, melanin formation appeared to be delayed by an additional day, with maximal A_492_ readings not reached until day 6. In contrast to HpaBC, melanin production was only observed in M9 medium, with no melanin production in LB. When examining protein expression levels, we could not clearly identify a distinguishably overexpressed band corresponding to MelA (predicted MW ∼55 kDa) (Supplementary Figure S2C). This indicated MelA is not expressed or tolerated well in *E. coli*, and perhaps the delayed production of melanin is a result of poor protein expression. Alternatively, the conditions tested may not be conducive for MelA activation (see discussion below).

In summary, Tyr1 was the most efficient melanizing enzyme expressed in *E. coli*, able to synthesize melanin in both M9 and LB media as well as demonstrating robust over-expression. HpaBC demonstrated good activity in LB, however, was unable to synthesize melanin in M9. This may be a result of inefficient translation or poor stability of the reductase component (HpaC), or potentially due to a limitation in available NADH/FADH_2_ required by this two-component system in a minimal medium. Lastly, MelA appears to be the least efficient enzyme, with melanin synthesis requiring 3–5 days of incubation and biosynthesis only occurring in M9 medium. Of the tested promoters, the constitutively expressed proteins in the pCB1D5 plasmid demonstrated a sustained and stable production of all enzymes tested. However, melanin production was strongest when expressed from the pJV plasmid when induced with IPTG. With these results in hand, we selected the pJV-Tyr1 plasmid to move forward with for testing recombinant protein expression and melanin biosynthesis in *V. natriegens*.

### 3.2 Development of defined minimal media for *V. natriegens* growth and melanin biosynthesis

Before expressing and initiating melanin production, a defined minimal media needed to be optimized to support robust growth when culturing *V. natriegens*. A previous study conducted by our lab demonstrated melanin production using the pJV-Tyr1 system in *V. natriegens* with LBv2 medium at a yield of ∼0.45 g/L (Wang et al., 2020). However, yields were significantly lower (∼0.05 g/L) when cultured in a minimally defined medium. Use of minimal media is highly desirable from a biomanufacturing perspective, where precise control over chemical compositions can significantly improve batch-to-batch variability, reduce costs typically associated with complex nutrient rich media mixes, permit selective culturing by omission/inclusion of specific nutrients, and allow for simplistic downstream processing and purification steps of the final product. Considering this, we sought to develop a defined minimal medium that would support both high density growth of *V. natriegens* and robust biosynthesis of melanin.

Standard M9 minimal medium typical for bacterial cell culturing with the addition of 1 mM MgSO_4_ and 0.1 mM CaCl_2_ was used as a starting point (Cold Spring Harbor Laboratory, 2010). As *V. natriegens* is a halophilic organism, additional sodium chloride was added to 200 mM. Concentrations of the individual components were then systematically varied and *V. natriegens* growth monitored by recording OD_600_ measurements in a microtiter plate format using a Bioscreen C over the course of 24 h. Endpoint analysis of OD_600_ and pH values of spent media were also analyzed. Results from these screens and identification of factors positively influencing *V. natriegens* growth are summarized in Table 2 and representative traces are depicted in Supplementary Figure S3. Ultimately, this screen allowed for the development of a minimal medium formulation specific for *V. natriegens* growth designated as VnM9v2 throughout the remainder of this manuscript.

**TABLE 2:** List of components tested and range of concentrations evaluated for the development of the various minimal media used in this study. Components highlighted in green significantly influenced *V. natriegens* growth. *V. natriegens* could not utilize sole carbon sources highlighted in red. * See methods for composition of 1,000X metals mix.

	Component	Range tested	Base	M9v2	M9v3	Comments
**Inorganic Salts**	Na/K Phosphate	0–200 mM	50 mM	100 mM	25 mM	Strong effect on growth and pH balancing
	Ammonium Chloride	0–100 mM	20 mM	50 mM	50 mM	
	Sodium Sulfate	0–50 mM	1 mM	5 mM	130 mM	
	Sodium Chloride	0–500 mM	200 mM	275 mM	-	Significant effect on growth
	Magnesium Chloride	0–25 mM	1 mM	1 mM	1	
	Calcium Chloride	0–10 mM	0.1 mM	0.3 mM	0.3	
**Carbon Source**	Glucose	0–60 mM	20 mM	20 mM	20 mM	Excellent growth as a sole carbon source
	Glycerol		-	0.4% (w/v)	0.4% (w/v)	
	Maltose		-	-	-	Excellent growth as a sole carbon source
	Trehalose		-	-	-	Excellent growth as a sole carbon source
	Sucrose		-	-	-	
	Lactose		-	-	-	Unable to use as sole carbon source
	Fructose		-	-	-	
	Xylose		-	-	-	Unable to use as sole carbon source
	Arabinose		-	-	-	
	Citrate		-	-	-	Unable to use as sole carbon source
	Malate		-	-	-	Excellent growth as a sole carbon source
	Succinate		-	-	-	
	Acetate		-	-	-	
**Amino Acids**	Casamino Acids	0%–1% (w/v)	-	0.2% (w/v)	0.2% (w/v)	Excellent growth as a sole carbon source
	Aspartate		-	0.2% (w/v)	0.2% (w/v)	Strong effect on pH balancing
	Glutamate		-	-	-	Strong effect on pH balancing
	18 amino acid mix		-	-	-	
**Other**	Metals Mix*	0–10X	-	0.2X	0.2X	Significant effect on growth
	Riboflavin	0–100 µM	-	-	-	
	Thiamine		-	1 µM	1 µM	
	Biotin		-	-	-	

Briefly, of the base salts tested, phosphate concentration had a significant effect on *V. natriegens* growth, presumably a result of the increased buffering capacity (Supplementary Figure S3A). Bacterial cultures respiring on glucose as a sole carbon source are known to generate and secrete organic acids, effectively dropping culture pH and arresting cellular growth. Phosphate concentrations dramatically effected final pH values, increasing from 4.8 to 7.3 over the concentration ranges tested, effectively allowing sustained growth and increased culture densities. Sodium chloride concentrations also significantly influenced growth (Supplementary Figure S3B). *V. natriegens* grew exceptionally well between 200 and 500 mM sodium chloride, highlighting the remarkable salt tolerance of this organism. Below 200 mM, growth was significantly delayed and/or reduced. Of the other inorganic salts tested, variations in their concentrations resulted in little to modest effects on growth.

We also screened various carbon sources for *V. natriegens* growth. As reported previously, *V. natriegens* is able to utilize a wide variety of carbon sources (Long et al., 2017; Ellis et al., 2019). Of the carbon sources tested, glucose, maltose, trehalose, and malate permitted the strongest growth profiles (Supplementary Figure S3C), with glucose maintained as the primary carbon source. We also screened combinations of carbon sources, specifically glucose and glycerol. Adding a secondary carbon source provides an alternative energy source for continued growth after cells have exhausted glucose. Additionally, metabolism of glycerol does not further contribute to the organic acid build up as respiration on glucose does, limiting the acidification of culture medium. Interestingly, supplementing with casamino acids (CAA) as the sole carbon source by far demonstrated the strongest growth profile. However, when supplementing with a pure mixture of individual amino acids, growth was only modest compared to glucose. CAA are typically impure peptide mixes, containing other impurities (including nutrients) carried over from the extraction process, and provides a likely explanation for this increased growth. This suggested other components within the CAA could be contributing to *V. natriegens* growth.

Lastly, we screened various supplements that influenced *V. natriegens* growth. Considering the strong positive effect CAA had as a sole carbon source, we screened low concentrations of CAA in the medium which still demonstrated a strong positive influence on growth. We also tested individual amino acids as supplements such as aspartate and glutamate, both of which positively influenced growth and significantly aided in buffering culture pH levels after overnight growth (Supplementary Figure S3D). These amino acids represent cheap alternative energy sources for cellular metabolism and can act as a metabolic buffer (Studier, 2005; Zampieri et al., 2019). Additionally, amino acids are known to act as osmolytes that may accumulate under conditions of osmotic stress (Sleator and Hill, 2002). The positive effect of aspartate supplementation may thus be three-fold: acting as an alternative energy source, balancing media pH, and serving as a protective osmolyte. Additional supplements tested included thiamine, biotin, and riboflavin, with only thiamine demonstrating a modest effect on growth. Lastly, the addition of a transition metal trace element mix had a very strong influence on *V. natriegens* growth (Supplementary Figure S3E). The addition of transition metals, especially iron, is well known to positively influence cellular growth (Studier, 2005; Andreini et al., 2008).

**FIGURE 5:**
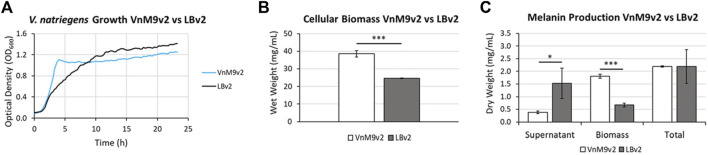
**(A)** Growth curves of *V. natriegens* in VnM9v2 vs. LBv2 recorded on a Bioscreen **(C)**. Traces represent averages of three biological replicates. **(B)** Measurement of *V. natriegens* cellular biomass in VnM9v2 vs. LBv2 media from 50 ml overnight shaking culture flasks. **(C)** Quantitation of melanin production of *V. natriegens* pJV-Tyr1 cells supplemented with 2 mg/ml tyrosine and 40 µM CuSO_4_. Biological triplicates were used for all studies. Error bars represent standard deviation of the mean. *p < 0.05; ***p < 0.005.

After establishing a recipe for VnM9v2 medium, growth was compared to traditional LBv2 medium. Overall, *V. natriegens* demonstrated exceptional growth in both media tested, however, cells grew much faster in M9v2, reaching stationary phase in ∼4 h compared to ∼10 h in LBv2 (Figure 5A). In terms of biomass alone, growth in VnM9v2 resulted in a ∼60% increase in total cellular biomass compared to LBv2 after overnight culturing (Figure 5B). Theoretically, increased biomass should lead to higher protein production and increased biosynthetic capacity. To test this, we induced Tyr1 expression in *V. natriegens* grown in both media and supplemented cultures with 2 g/L tyrosine to initiate melanin biosynthesis. Cultures were incubated overnight and the following morning cultures were harvested. Culture containing supernatants had the characteristic black color of melanin (“supernatant melanin”). However, it was also noted that pelleted cells were distinguishably black, particularly in the VnM9v2 medium, suggesting the presence of intracellular or cell-associated melanin (“biomass melanin”). These two melanin sources were initially kept separate, and subjected to three rounds of acid-base cycling to purify the melanin (Supplementary Figure S4). Interestingly, depending on the media used, opposite trends in melanin localization were seen. In LBv2 medium, roughly 2/3 of the melanin was isolated from culture supernatants, while roughly 2/3 of the melanin was associated with cell biomass in VnM9v2 (Figure 5C). This may be likely due to the increased viability of cells in the optimized VnM9v2, whereas increased cell death and lysis in LBv2 medium results in increased release of Tyr1 into culture supernatants. However, when combined, the total melanin generated in VnM9v2 and LBv2 were essentially identical at 2.19 g/L, representing an estimated ∼103% product conversion rate based on mass conversion of tyrosine to a DHICA monomer subunit.

### 3.3 Protein expression and melanin biosynthesis in *V. natriegens*

**FIGURE 6:**
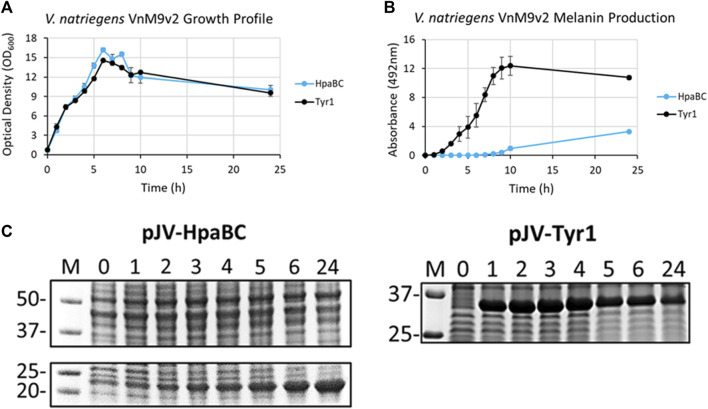
*V. natriegens* cultures in VnM9v2 medium expressing either Tyr1 (black traces) or HpaBC (blue traces) in the pJV plasmid after induction with IPTG and supplementation with tyrosine and CuSO_4_ (for Tyr1 only). **(A)** OD_600_ culture density measurements taken from control cultures not supplemented with tyrosine. **(B)** Melanin production by measuring A_492_. Measurements were taken from cell-free culture supernatants after centrifugation. Readings were taken on biological triplicates. **(C)** Protein expression profiles of HpaBC and Tyr1 after induction with 1 mM IPTG. Values above each lane represent hour post-induction.

After selection of Tyr1 as the most efficient melanizing enzyme and the development of a fully defined VnM9v2 minimal medium, the pJV-Tyr1 plasmid was transformed into *V. natriegens* to compare protein expression and melanin induction profiles in this emergent chassis organism. While we focus on Tyr1 throughout the remainder of this manuscript, to be rigorous we also examined HpaBC expression and melanin induction. Growth profiles were similar in VnM9v2 for expression of either Tyr1 or HpaBC (Figure 6A). Noticeably, maximum OD_600_ values were over 3 times higher in optimized VnM9v2 medium than for *E. coli* grown in minimal media from earlier experiments (Figure 3A and Figure 4A). As seen previously, there was efficient melanin production in Tyr1 expressing cells, with maximal melanin production plateauing around 9 h post-induction (Figure 6B). In contrast to *E. coli*, there was evidence of melanin production in VnM9v2 with the HpaBC enzyme system, albeit significantly less efficient compared with Tyr1 (Figure 6B). Melanin production was not apparent until almost 10 h post-induction, and maximal A_492_ readings were over 3 times lower than for Tyr1. This again validated our selection of Tyr1 as the most efficient melanizing enzyme in minimal medium.

To assess protein expression levels, cell extracts at the indicated time points were separated and analyzed by SDS-PAGE. Protein expression profiles were similar between *E. coli* and *V. natriegens* using the pJV plasmid. There was a strong and rapid appearance of both Tyr1 and HpaBC after induction with 1 mM IPTG, with sustained protein levels throughout the course of the experiment (Figure 6C). Of note, maximal protein expression levels in *V. natriegens* occurred after 2 h of incubation, whereas maximal protein levels appeared to occur after 5 h in *E. coli* (Figure 3C and Figure 4C). This is likely due to the increased ribosome content reported in *V. natriegens* (Aiyar et al., 2002), allowing for robust and rapid protein synthesis. Also of interest was the fact that HpaC appeared to be stably expressed in *V. natriegens* as evidenced by an overexpressed band ∼20 kDa, which was contrary to what was seen in *E. coli*. Regardless, despite sufficient expression of HpaBC, melanin production significantly lagged behind Tyr1, again hinting at insufficient HpaBC activity or an insufficient oxidizing environment to convert L-DOPA to melanin. For the remainder of this manuscript we choose to focus on optimizing melanin production in *V. natriegens* using the pJV-Tyr1 system, which demonstrated the most robust and efficient protein expression and melanin biosynthetic profile in VnM9v2 medium.

### 3.4 Optimizing and improving melanin biosynthesis in *V. natriegens*

Initial analysis of melanin biosynthesis in *V. natriegens* resulted in complete product conversion rates (Figure 5C), indicating melanin yields were limited by substrate concentration. Rationally, increasing substrate concentration (tyrosine) should lead to increased product yields (melanin). To test this, *V. natriegens* with pJV-Tyr1 was grown to log phase in VnM9v2 medium and protein production induced by IPTG. Cells synthesized Tyr1 for 3 h before addition of CuSO_4_ and increasing concentrations of tyrosine to initiate melanin biosynthesis. The biosynthetic phase proceeded overnight at 37 °C.

**FIGURE 7:**
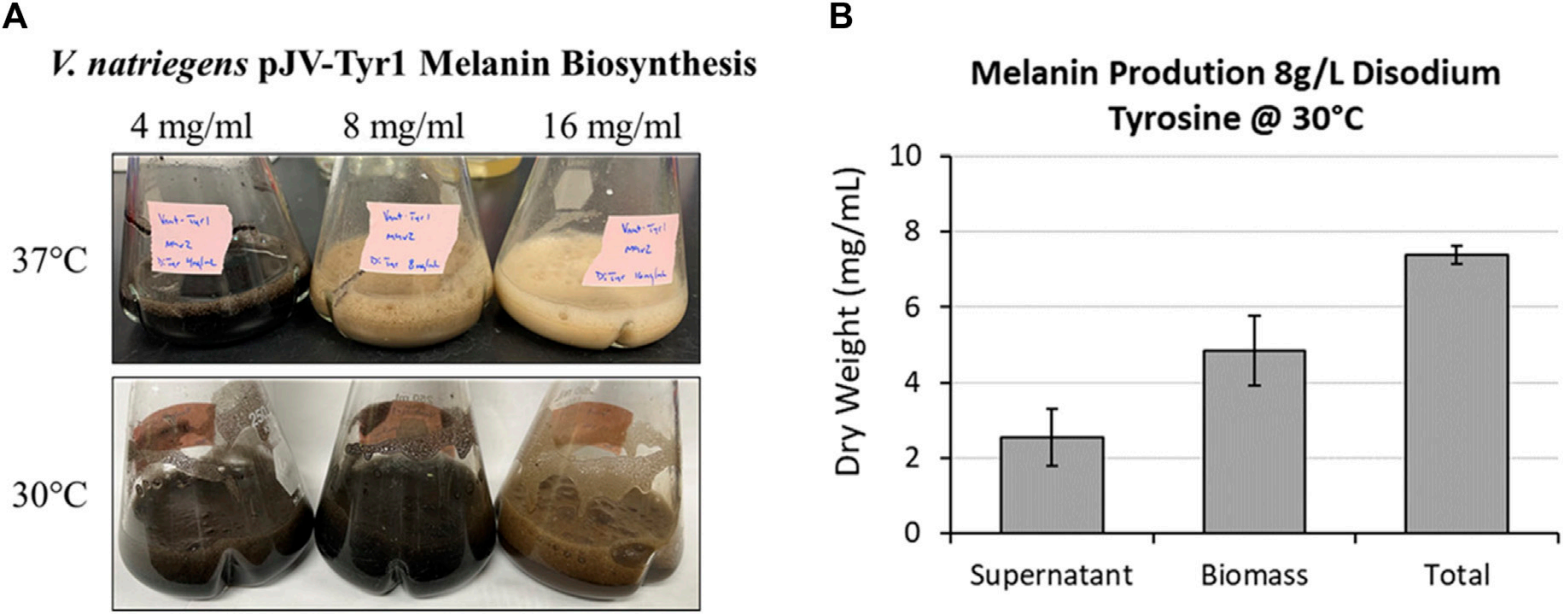
Using disodium tyrosine as a substrate significantly increased melanin biosynthetic yields due to increased substrate solubility. **(A)** Images of overnight expression cultures of *V. natriegens* supplemented with the indicated concentrations of disodium tyrosine and incubated at either 37 °C (top) or 30 °C (bottom). Black pigmentation is indicative of melanin biosynthesis. **(B)** Quantification of melanin extracted from *V. natriegens* cultures supplemented with 8 g/L disodium tyrosine and incubated at 30 °C. Average total yields were 7.40 g/L. Representative of three biological replicates. Error bars represent standard deviations of the mean.

As seen in the previous experiment (Figure 5C), melanin biosynthesis reached near 100% product conversion rates when supplemented with 2 g/L tyrosine. However, at higher concentrations, no melanin formation was observed, and tyrosine added to cultures remained as a white precipitate in solution. This could be due to toxicity issues, substrate inhibition of enzyme activity, or issues with substrate solubility. To counter the inherent solubility issues, tyrosine was replaced with the disodium salt form which has >100-fold increase in solubility due to the additional charge provided and the disruption of clustering of non-polar aromatic rings. Repeating the same experiment with disodium tyrosine resulted in increased melanin biosynthetic yields, able to synthesize melanin using tyrosine concentrations up to 4 g/L (Figure 7A). However, melanin biosynthesis was again inhibited at substrate concentrations above 4 g/L. It had been previously noted that melanin yields using recombinant MelA expressed in *E. coli* were significantly improved by reducing reaction temperatures from 37 °C to 30 °C (42, 51). Similarly, when reaction temperatures were reduced to 30 °C, Tyr1 expressing cultures were able to synthesize melanin at substrate concentrations of 8 g/L (Figure 7A). On average, overnight cultures supplemented with 8 g/L disodium tyrosine resulted in production yields of 7.40 g/L melanin (∼107% product conversion rate) (Figure 7B). This equates to one of the highest reported volumetric productivities to date for the biomanufacture of melanin at roughly 463 mg L^−1^ h^−1^. Also similar to previous experiments, the majority of melanin was associated with cellular biomass, with the remainder isolated from culture supernatants (Figure 7B). At substrate concentrations above 8 g/L, melanin biosynthesis was observed, but product yields were significantly reduced or melanin formation was completely inhibited, indicating again potential issues with substrate solubility or enzyme inhibition (Figure 7A). This significant improvement in yield and volumetric productivity is a promising development for economical, industrial scale biomanufacturing of melanin.

We also tested melanin toxicity as a plausible explanation for the plateauing of melanin biosynthesis around 8 g/L. After a 2 h incubation of *V. natriegens* cultures with increasing concentrations of melanin, we saw a slight, but non-significant dose-dependent reduction in bacterial colony forming units (CFUs) up to 10 g/L melanin (Supplementary Figure S5). At the highest concentration tested (10 g/L melanin) there were still >10^8^ CFU/ml viable *V. natriegens* cells. For comparison, we also tested melanin toxicity with *E. coli* and saw no significant reductions in CFUs with increasing concentrations of melanin, up to 10 g/L. While there was a slight decreasing trend in viable cell counts for *V. natriegens* with increasing melanin concentrations, this could be due to slight melanin toxicity or reductions in bacterial cell growth. Either way, at concentrations >10^8^ CFU/ml, the slight toxicity or reduction in growth is not likely the cause for inhibited melanin production above 8 g/L, and is more likely reflective of the low solubility of tyrosine.

### 3.5 Transitioning melanin biosynthesis into small-scale bioreactors

The developed VnM9v2 medium in this study facilitated superior *V. natriegens* growth and melanin biosynthesis in shaking culture flasks. For industrial scale biomanufacturing, large 100–1000 L biofermentors are typically preferred. However, there are several factors which have to be considered when moving the bioprocess to a bioreactor. A key concern of culturing *V. natriegens* in a bioreactor system is corrosion of metallic bioreactor components (vessels, impellers, tubing, flanges, *etc.*) during prolonged and repeated exposure to high salt medium. Additionally, with automatic pH sensing and real time adjustments with acid/base titration, the high buffering capacity (100 mM) of VnM9v2 is unnecessary. Reduction in buffering capacity would additionally reduce the amount of acid/base required for pH maintenance. Lastly, reduction in phosphate concentration would translate to significant cost savings for large scale manufacturing. With these considerations in mind, a variation of the VnM9v2 medium was needed to accommodate transition of *V. natriegens* cultivation into a bioreactor format.

First, the halophilic nature of *V. natriegens* requires elevated salt concentrations for optimal growth. In VnM9v2 medium, 275 mM sodium chloride is provided to support this requirement. Specifically, monoionic ions like sodium (Na^+^) are required for optimal growth, while it is the chloride ion (Cl^−^) that presents bioreactor fouling and corrosion issues. Thus, substitution of NaCl with an alternative sodium salt with a non-corrosive counter-ion should sustain *V. natriegens* growth while alleviating bioreactor corrosion concerns. This was first tested by substituting NaCl with Na_2_SO_4_ in VnM9v2 medium, making sure to maintain equivalent Na^+^ ion concentrations (∼275 mM). *V. natriegens* cells were inoculated into VnM9v2 medium and the Na_2_SO_4_ substituted version in 100 ml bioreactor vessels. Real-time monitoring of optical densities using a spectrophotometric probe revealed very similar growth profiles between the original VnM9v2 and Na_2_SO_4_ substituted variant (Supplementary Figure S6). Second, reducing phosphate levels to obviate the need for large volumetric additions of acid/base to maintain stable pH values near neutral during a bioreactor run were evaluated. Using the small scale bioreactor system again, 100 ml cultures were set up using standard VnM9v2 or phosphate reduced media. From these runs, lowering phosphate levels to 20 mM resulted in very similar growth trends to VnM9v2 medium while maintaining culture pH (Supplementary Figure S6).

**FIGURE 8:**
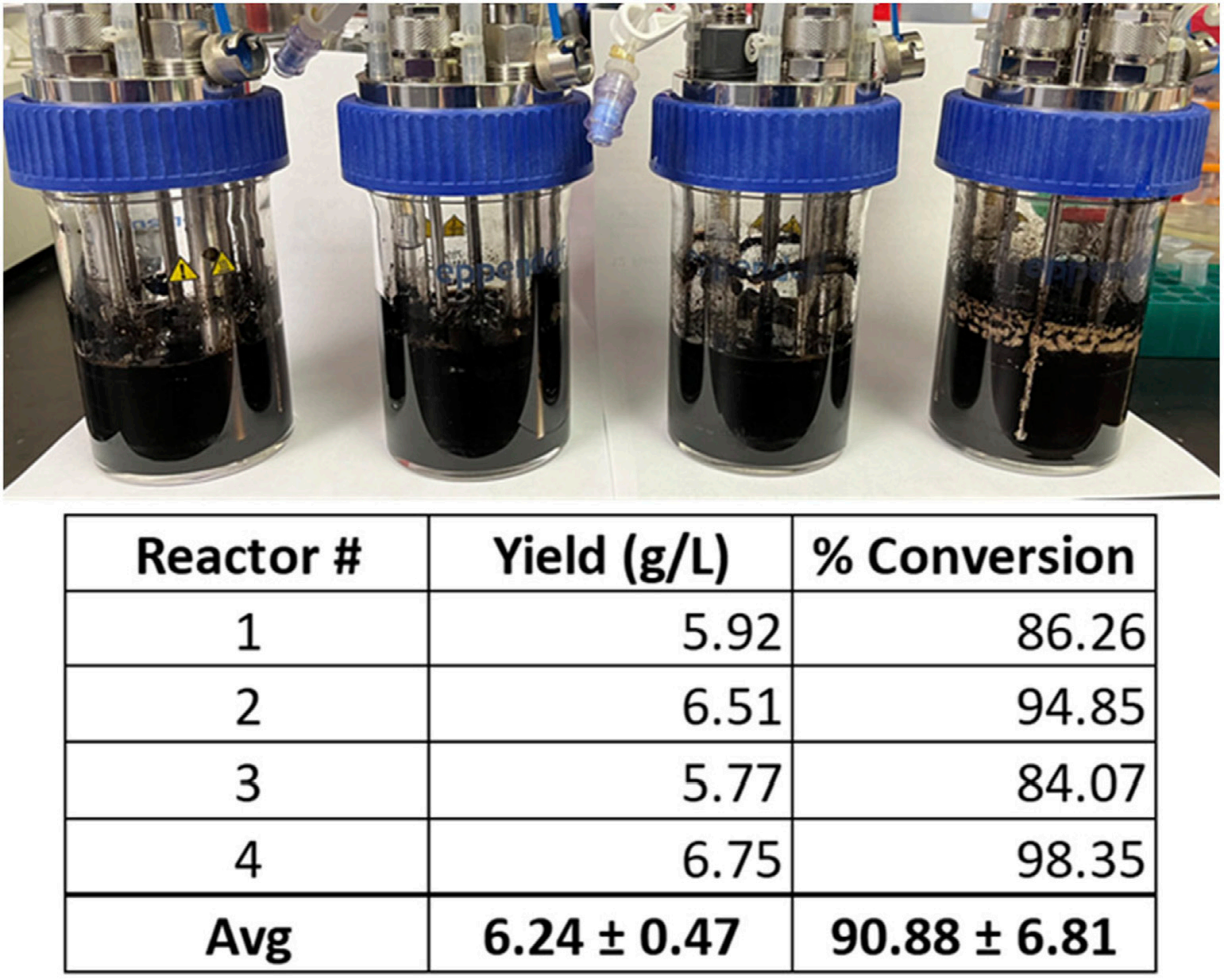
*V. natriegens* biosynthesis of melanin in 100 ml VnM9v3 medium using small-scale bioreactors. Reported yields and product conversion rates are listed below for each reactor.

From these experiments, the modified VnM9v3 was formulated specific for bioreactor growth of *V. natriegens* (Table 2). Lastly, using the newly developed VnM9v3, melanin biosynthesis was tested in bioreactors. Cultures were similarly grown at 37 °C to log phase, after which the temperature was lowered to 30 °C and Tyr1 expression induced with 1 mM IPTG for 3 h. After induction, 40 µM CuSO_4_ and 8 g/L disodium tyrosine was added. Cultures were incubated overnight and collected the following morning. All reactors were visibly black, indicative of melanin biosynthesis using VnM9v3 medium in bioreactors (Figure 8). Extraction and purification of melanin resulted in total product yields averaging 6.24 g/L, roughly a 91% product conversion rate, corresponding to a productivity of 390 mg L^−1^ h^−1^ (Figure 8). While slightly less efficient than VnM9v2 medium in shake culture flasks which reached near 100% product conversion rates, there is still much room for improvement by tuning bioreactor operating conditions, such as dissolved oxygen content, agitation speeds, pH adjustments, and temperature control. Additionally, fed-batch or continuous bioreactor setups could be explored to improve biosynthesis.

### 3.6 Identification of a copper inducible promoter in *V. natriegens*

Inducible control of protein expression using the *lac* promoter and IPTG is a well-established and strong regulator of protein expression in bacteria (Shilling et al., 2020). It has been adopted as a wide-spread standard for laboratories recombinantly expressing proteins in *E. coli*. Similarly, this system has been adopted in *V. natriegens* (Weinstock et al., 2016; Tschirhart et al., 2019; Carrillo Rincon and Farny, 2023). However, a significant drawback of this system is its tunability and leaky expression (Rosano and Ceccarelli, 2014), with even small doses of IPTG resulting in dramatic gene expression, but more so for large scale biomanufacturing is the steep cost of using IPTG as an inducing molecule. In this respect, we sought to identify alternative induction systems compatible in *V. natriegens* that would respond to a different chemical signal to initiate melanin biosynthesis.

**FIGURE 9:**
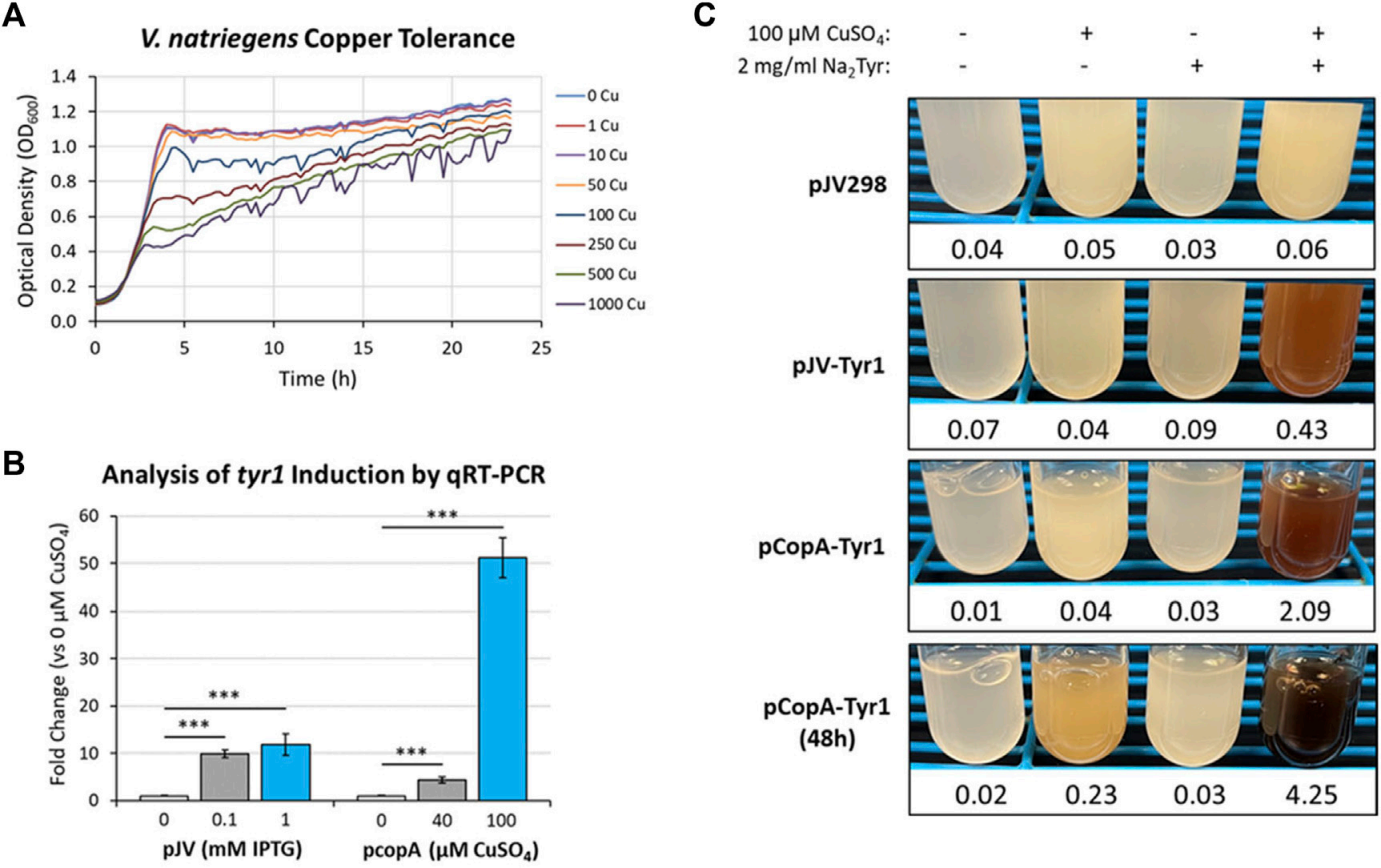
**(A)** Bioscreen C growth curves of *V. natriegens* cultures in VnM9v2 medium supplemented with increasing concentrations of CuSO_4_. **(B)** qRT-PCR analysis of *tyr1* transcript induction using the indicated concentrations of IPTG or CuSO_4_ for the pJV-Tyr1 or pCopA-Tyr1 plasmids, respectively. Transcript abundances were normalized to the housekeeping gene *gapA*. Error bars represent standard deviations of the mean. ***p < 0.005. **(C)** Copper induction and melanin biosynthesis with the *copA* promoter. *V. natriegens* cultures containing the indicated plasmids were supplemented with 100 µM CuSO_4_ and/or 2 mg/ml disodium tyrosine (Na_2_Tyr) as indicated and incubated overnight, or up to 48 h for the pCopA-Tyr1 strains. Values below each culture represent absorbance values at 492 nm from cell free culture supernatants for measurement of melanin formation.

One such response that has been well documented in microbes is metal toxicity, particularly for copper (Smith et al., 2017; Giachino and Waldron, 2020). Many microbes have evolved sensitive and sophisticated mechanisms for sensing and detoxifying excess copper encountered in the environment. Using a bioinformatics approach and manual curation of the *V. natriegens* genome, we screened for known and putative homologous genes related to copper homeostasis/detoxification which identified several candidate genes (Supplementary Table S3). However, before testing for copper induction, the copper tolerance of *V. natriegens* needed to be assessed to determine appropriate concentrations for triggering a copper stress response. Cultures were grown in VnM9v2 supplemented with increasing concentrations of CuSO_4_ and growth monitored over the course of 24 h (Figure 9A). *V. natriegens* grew exceptionally well up to concentrations of 50 µM CuSO_4_ and were similar to unsupplemented control cultures. This result was promising, as we routinely supplement cultures with 40 µM CuSO_4_ to supply copper for Tyr1 activity. However, beginning at 100 µM CuSO_4_ cultures began to demonstrate growth deficiencies, with the severity intensifying as copper concentrations continued to increase. Surprisingly, *V. natriegens* demonstrated remarkable copper tolerance, with cultures still exhibiting growth when challenged at the highest concentration tested of 1 mM CuSO_4_, albeit significantly attenuated compared to control cultures. To minimize toxicity issues, yet stimulate a copper stress response, we choose the concentration of 100 µM CuSO_4_ for subsequent studies.

Next, primers were designed for genes identified in the bioinformatics screen (Supplementary Table S3) and their copper induction was evaluated by qRT-PCR. One of the highest induced genes in response to copper was the cytosolic copper exporter *copA* (Supplementary Figure S7), established as a primary copper detoxifying protein in several Gram-negative bacteria (Outten et al., 2000; Rensing et al., 2000). The promoter of this gene was selected and cloned into the pJV backbone, replacing the *Ptac* and *LacI* ^*Q*^ elements, for regulation of the *tyr1* gene. A promoter fragment corresponding to ∼200 base pairs upstream of the start codon was selected as the most optimal promoter region for gene regulation. *V. natriegens* cultures harboring the newly constructed pCopA-Tyr1 plasmid were grown in VnM9v2 medium to logarithmic phase and challenged with either 40 µM or 100 µM CuSO_4_ for 3 h. In parallel cultures, cells harboring pJV-Tyr1 plasmid were also assessed for *tyr1* induction using IPTG as a comparison. Subsequent RNA was isolated and induction of *tyr1* transcript levels were assessed by qRT-PCR (Figure 9B). Induction of tyr1 with the pJV-Tyr1 plasmid resulted in ∼10- and 12-fold induction of transcript levels when induced with 0.1 mM and 1 mM IPTG, respectively. Comparatively, with the pCopA-Tyr1 plasmid, *tyr1* levels were induced ∼5-fold when supplemented with the relatively non-toxic level of 40 µM CuSO_4_. However, cultures supplemented with 100 µM CuSO_4_ demonstrated a robust >50-fold induction in *tyr1* transcript levels, suggesting strong activation of the copper stress response and is in good agreement with the previous growth data (Figure 9A).

Lastly, we tested the ability of cells harboring the pCopA-Tyr1 plasmid to induce melanin biosynthesis when activated with copper. As controls, we also included cultures harboring the empty pJV298 or pJV-Tyr1 plasmids. Cultures were then supplemented with and/or without 100 µCuSO_4_ and 2 mg/ml disodium tyrosine and allowed to incubate for 24 h at 30 °C. As seen in Figure 9C, cultures harboring the pCopA-Tyr1 plasmid demonstrated the ability to synthesize melanin when supplemented with copper and tyrosine, albeit not quite as well as the pJV-Tyr1 cells when induced with IPTG (Figure 6B). Extending the incubation to 48 h resulted in further melanin formation, indicating a sustained promoter and/or enzyme activity. Importantly, the tight regulation of this promoter system was evident by the lack of any melanin formation in cultures supplemented with substrate tyrosine only. Conversely, control cultures harboring the pJV-Tyr1 plasmid demonstrated low levels of melanin biosynthesis when supplemented with copper and tyrosine, despite no addition of IPTG, highlighting the leaky nature of this promoter system. The empty pJV298 control plasmid demonstrated no visible melanin formation under any condition tested, indicating there was no auto-oxidation of tyrosine in the presence of high levels of copper, and that melanin formation was indeed synthesized catalytically via Tyr1. Thus, our current *copA* promoter construct may serve as a weakly inducible system with very tight control over basal gene expression. Further characterization of the *copA* promoter is underway for further development and optimization of this system for use in *V. natriegens*.

## 4 Discussion

The unique physicochemical properties of melanin have garnered significant interest from the biotechnology field for the development of novel melanin-based biomaterials spanning a wide variety of potential applications. However, the realization of economical, industrial scale biomanufacturing of melanin using microbes has been stifled by low yields and/or poor volumetric productivities. For example, cultivation of *Klebsiella* sp. (Sajjan et al., 2010), *B. subtilis* (Ghadge et al., 2020), and *Auricularia auricula* (Sun et al., 2016) only resulted in reported total yields ranging from 0.13–2.96 g/L. Identification and cultivation of new melanizing microbes, genetic engineering efforts, or recombinant protein expression have led to improvements on this front. For example, cultivation of *Streptomyces sp*. ZL-24 in soy peptone medium allowed for production capacities of 4.24 g/L (Wang et al., 2019). Another group used *Pseudomonas koreensis* in a molasses and tyrosine medium to obtain melanin yields of 5.5 g/L (Eskandari and Etemadifar, 2021). Even further, yields of 6 g/L were reported in *E. coli* recombinantly expressing a mutant variant of MelA when batch-fed with tyrosine as a substrate (Lagunas-Munoz et al., 2006). While these product yields are significant improvements, they required multiple days of incubation resulting in poor to suboptimal volumetric productivities. By far one of the most significant yields reported in the literature to date was accomplished using the natural melanin producing fungus *Armillaria cepistipes* (Ribera et al., 2019). An impressive 28 g/L melanin was obtained when supplementing cultures with tyrosine as a substrate. However, this required an extensive incubation period of 161 days resulting in a very poor productivity of 174 mg L^−1^ d^−1^. To date, one of the highest reported efficiencies was accomplished using the bacterium *Streptomyces kathirae* expressing the enzyme MelC (Guo et al., 2015). This system was also able to produce impressive melanin titers of 28 g/L, but only required a 5 days incubation period, equating to a significant improvement in volumetric productivity of 225 mg L^−1^ h^−1^. However, a major limitation to this approach was the requirement of excessive amounts of yeast extract in the medium, a cost prohibitive component for industrial scale manufacturing. Additionally, aromatic components within complex nutrient mixes can serve as a substrate for tyrosinase enzymes that are incorporated into the melanin biopolymers, introducing impurities and further chemical heterogeneity to the final melanin product. In this study, we were able to achieve maximal production yields of 7.4 g/L with an efficiency of 473 mg L^−1^ h^−1^. Calculated substrate to product conversion rates varied from 97%–107%. Melanin is well documented to chelate monionic and divalent metals and is a likely cause for the discrepancies of the increased efficiency above 100%, and recently published XPS data support this hypothesis (Dysart et al., 2023).

In this study, three enzymes were recombinantly expressed and screened for their ability to catalyze the oxidation of tyrosine to melanin in both *E. coli* and *V. natriegens*. We found the tyrosinase Tyr1 to be the most efficient melanizing enzyme, particularly when expressed in minimal medium, able to biosynthesize melanin at titers of 7.4 g/L in *V. natriegens*. One significant advantage of using *V. natriegens* as a host chassis is its rapid growth and ability to reach significantly higher culture densities in our optimized VnM9v2 medium compared to *E. coli*. Additionally, maximal protein expression of Tyr1 occurred much sooner in *V. natriegens* (2 h) compared to *E. coli* (5 h), allowing for a faster transition from the induction phase to the biosynthetic phase. In contrast, HpaBC demonstrated strong activity, but only in nutrient rich medium. In minimal medium, it appeared HpaC was unstable in *E. coli*, given the lack of visible expression by SDS-PAGE. However, V. natriegens was able to stably express HpaC in minimal medium, again highlighting the exceptional protein expression capacity of this organism. However, melanin biosynthesis was still impaired, and may be due to an insufficiency of required NADH/FADH2 cofactors. Alternatively, HpaBC is only capable of the initial oxidations to generate L-DOPA, and our results suggest the minimal medium tested present an insufficient oxidizing capacity for subsequent auto-oxidation of L-DOPA to melanin (Supplementary Figure S1 **)**. As mentioned previously, complex nutrient rich media is not desirable from an industrial manufacturing perspective due to increased costs and incorporation of impurities into the final melanin product. Supplementation of cultures in with NADH and FADH_2_ in minimal medium was also not attempted as these cofactor costs would be prohibitive for large scale manufacturing. For these reasons, HpaBC was not considered an optimal enzyme to use under the current conditions.

Interestingly, the tyrosinase MelA demonstrated very little activity, requiring roughly 5–6 days of incubation before maximal melanin production was observed in *E. coli*. Upon further examination of the protein sequence, MelA may represent a member of the class III tyrosinases which is expressed as a zymogen, requiring proteolytic processing of an extended C-terminal domain for enzyme activation (Fairhead and Thony-Meyer, 2010; Fairhead and Thony-Meyer, 2012). This C-terminal domain is proposed to interact closely with the surface of the catalytic active site, where a key tyrosine residue is positioned within the active site pocket to prevent substrate diffusion and catalytic activity. While MelA has been previously used to synthesize melanin, it has been reported to have an initial lag phase before melanin production is observed, typically not initiating melanin biosynthesis until stationary phase (Cabrera-Valladares et al., 2006). A mutant version of MelA has also been reported (mutMelA) where a single point mutation (Asp535Gly) in the C-terminal domain results in earlier initiation of melanin biosynthesis compared to the wild type enzyme (Lagunas-Munoz et al., 2006). While nowhere near the active site, this mutation may disrupt the C-terminal interaction with the active site or enhance proteolytic processing of this domain. In this study, the significant lag in melanin production is likely due to insufficient proteolytic processing of MelA in *V. natriegens* or the protease deficient *E. coli* BL21 (DE3) strain. To our knowledge, the role of the C-terminal domain in *R. etli* MelA has not been investigated. Currently, studies are underway utilizing a series of C-terminally truncated MelA variants to evaluate effects on enzyme activity and possibly engineer a more efficient version of this enzyme.

In our studies, expression of Tyr1 was strongest with IPTG induction in the pJV plasmids *versus* the constitutive pCB1D5 plasmids. However, IPTG is not desirable for scale-up manufacturing or expression of toxic proteins due to its high cost and leaky expression profile. Ideally recombinant protein expression is precisely controllable and tunable, preferably during the biosynthetic phase. This allows for cellular resources to be devoted to growth during early stages of the reactor process to achieve maximal cellular biomass before over-expression of a desired heterologous protein and subsequent biosynthesis of a desired product. With this in mind, we screened and identified an alternative endogenous inducible promoter responding to copper. Copper induction has been previously used in controlling Cas9 expression in the plant *Nicotiana benthamiana* using the copper responsive transcription factor CUP2 and a minimal promoter containing a Copper Binding Site (CBS) (Garcia-Perez et al., 2022). Copper regulation has also been reported in the opportunistic pathogenic yeast *Cryptococcus neoformans*, albeit control of heterologous gene expression was in response to copper starvation rather than copper toxicity using the copper importer *CTR4* promoter (Ory et al., 2004). In *V. natriegens*, preliminary analysis identified *copA* to be the highest regulated gene tested in response to excess copper (100 µM), eliciting a 50-fold induction of *tyr1* transcript levels. In comparison, IPTG induction of *tyr1* in the pJV plasmid only demonstrated a 10-fold induction. While absolute values of transcript levels cannot be directly compared in this manner, copper induction shows a ∼5-fold greater dynamic control of gene expression, possibly representing tighter control and reducing “leaky” expression levels compared to IPTG induction. This was further evidenced by the formation of melanin in copper and tyrosine supplemented pJV-Tyr1 cultures in the absence of IPTG, whereas there was no apparent melanin formation in pCopA-Tyr1 expressing cultures in the absence of copper (Figure 9C). This tight level of regulation with copper represents a promising alternative to traditional *lac* promoter systems and can be introduced as an additional resource for the *V. natriegens* molecular toolbox. This is especially desirable for precise induction of toxic proteins or enzymes that catalyze the formation of toxic products to initially maximize cellular growth and curtail toxicity. Further characterization of the *V. natriegens copA* promoter is underway to identify key promoter elements and regions to further enhance the utility and activity of this promoter system for heterologous protein expression.

Lastly, we transitioned our system from shaking culture flasks to small scale bioreactors to simulate conditions for industrial manufacturing. The development of VnM9v3 medium was critical to prevent long-term bioreactor fouling and corrosion when using high salt medium required for optimal *V. natriegens* growth. Substitution of sodium chloride with the less corrosive sodium sulfate demonstrated comparable growth and permitted melanin production in a bioreactor system, albeit at slightly reduced product conversion rates (∼91%). Conditions were kept consistent throughout the bioreactor runs, and refinement of bioreactor process parameters could lead to improvements in biosynthetic yields and reaction efficiencies. Efforts are underway to explore the effects dissolved oxygen, pH, agitation speed, and temperature have on production yields and efficiencies. In particular, dropping reaction temperatures had a significant effect on total reaction yields (Figure 7), and it will be interesting to see if further improvements can be gained by optimization of bioreactor processes. In this study, we only evaluated a single batch process, but optimizing a fed-batch process can lead to higher biomass production, and potentially higher product yields. Additionally, setting up a slow, steady feed of substrate could help overcome substrate toxicity, substrate solubility, and/or enzyme inhibition. The influence many of these process parameters have on *V. natriegens* growth have recently been evaluated and can serve as a blueprint moving forward for optimizing melanin production (Thiele et al., 2021). Further, cost savings can be gained by using an alternative promoter system, such as the *copA* promoter, or genomic integration of Tyr1 into the chromosome of *V. natriegens*, which would preclude the use of costly IPTG and antibiotics, respectively.

In conclusion, using an optimized VnM9v2 minimal medium we were able to biosynthesize melanin by recombinantly expressing the tyrosinase Tyr1 in the fast growing marine bacterium *V. natriegens*. Supplementing cultures with disodium tyrosine as a substrate achieved maximal product yields of 7.57 g/L from overnight cultures, representing a minimum production efficiency of 473 mg L^−1^ h^−1^. This is one of the highest reported volumetric productivities we could find reported in the literature to date. This value likely represents an underestimate as cultures were allowed to proceed overnight (∼16 h) and initial studies (Figure 4) as well as a previous report indicate melanin production may reach completion in as early as 10 h (Wang et al., 2020). Further, we developed a refined VnM9v3 medium for transition into bioreactors and demonstrated comparable growth and production efficiencies, representing a promising economical solution for scale-up biomanufacturing of melanin. Currently, plans are underway for transitioning and testing this system in industrial 100–1000 L biofermentors for the biomanufacture of melanin at the kilogram scale. Additionally, technoeconomic analysis will be conducted for the current materials, melanin purification, and downstream processing steps required to estimate the cost for its industrial manufacturing.

## Data Availability

The original contributions presented in the study are included in the article/Supplementary Material, further inquiries can be directed to the corresponding author.
