# Supplementary material for: Rapid, high-titer biosynthesis of melanin using the marine bacterium Vibrio natriegens
Source: Front Bioeng Biotechnol. 2023 Sep 13;11:1239756. doi: 10.3389/fbioe.2023.1239756 (PMC10534004; doi:10.3389/fbioe.2023.1239756)
Supplement: Supplementary file 3 [file Presentation1.PPTX]

## Slide 1
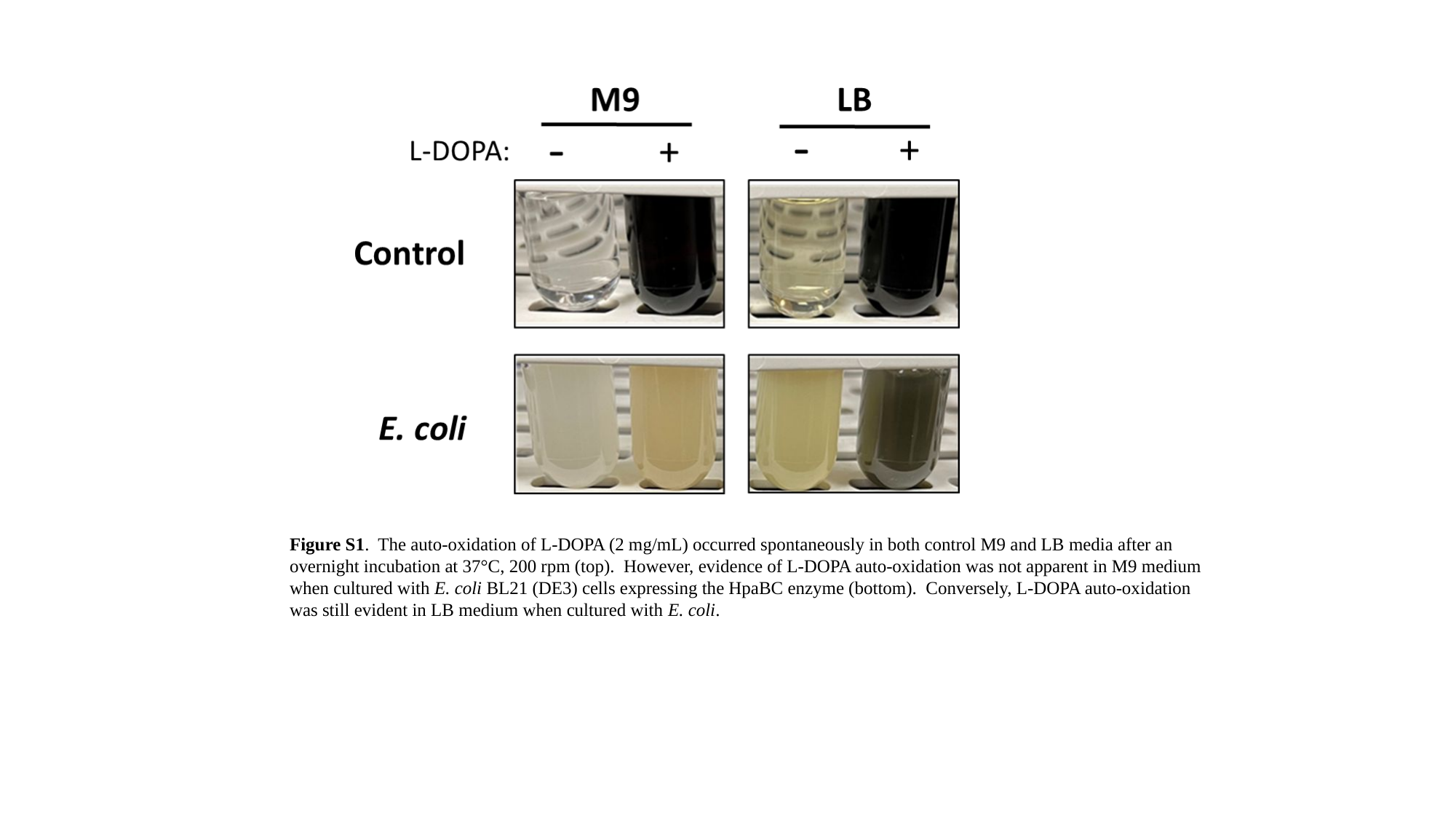

Figure S1. The auto-oxidation of L-DOPA (2 mg/mL) occurred spontaneously in both control M9 and LB media after an overnight incubation at 37°C, 200 rpm (top). However, evidence of L-DOPA auto-oxidation was not apparent in M9 medium when cultured with E. coli BL21 (DE3) cells expressing the HpaBC enzyme (bottom). Conversely, L-DOPA auto-oxidation was still evident in LB medium when cultured with E. coli.

## Slide 2
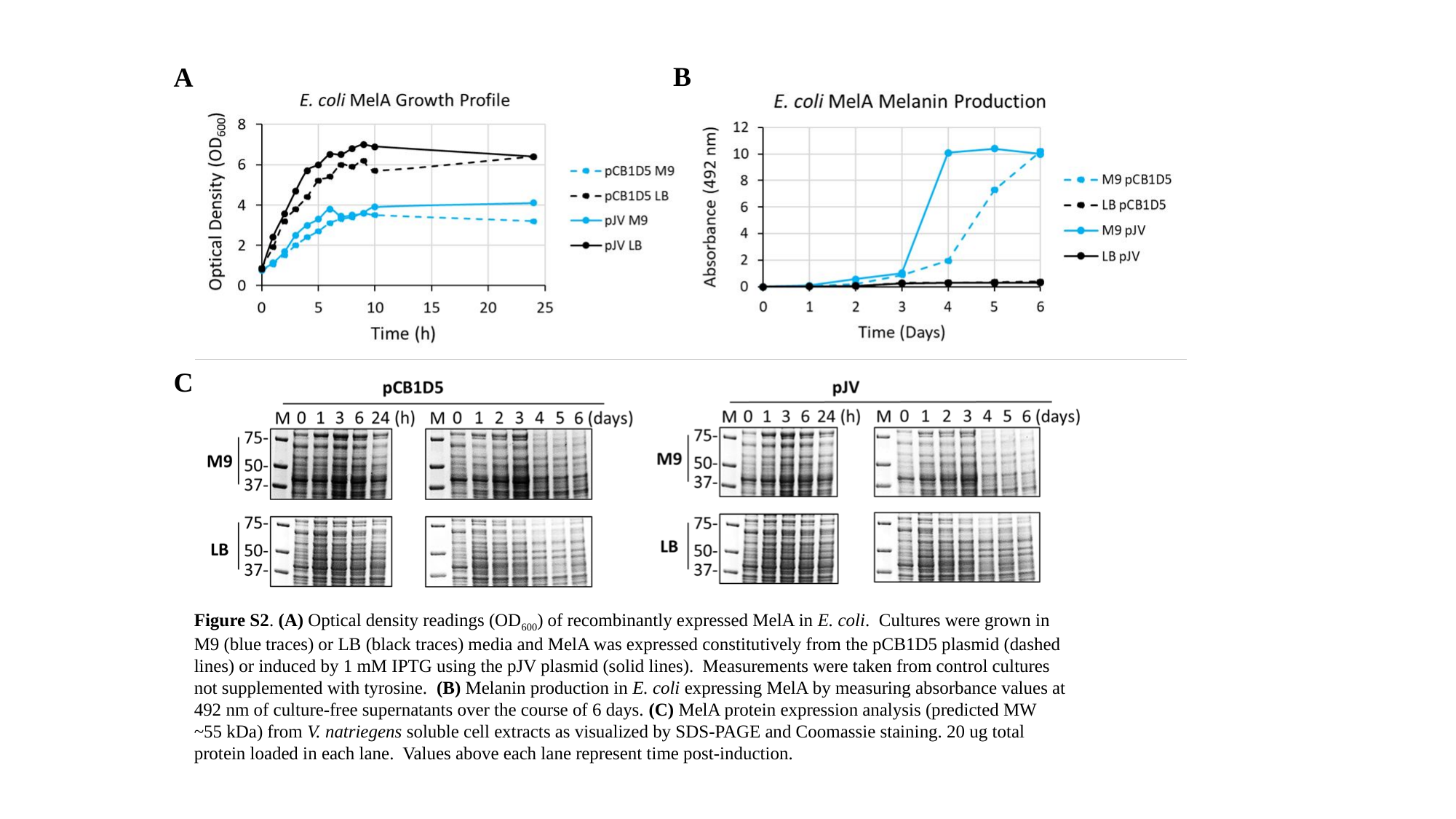

B
A
C
Figure S2. (A) Optical density readings (OD600) of recombinantly expressed MelA in E. coli. Cultures were grown in M9 (blue traces) or LB (black traces) media and MelA was expressed constitutively from the pCB1D5 plasmid (dashed lines) or induced by 1 mM IPTG using the pJV plasmid (solid lines). Measurements were taken from control cultures not supplemented with tyrosine. (B) Melanin production in E. coli expressing MelA by measuring absorbance values at 492 nm of culture-free supernatants over the course of 6 days. (C) MelA protein expression analysis (predicted MW ~55 kDa) from V. natriegens soluble cell extracts as visualized by SDS-PAGE and Coomassie staining. 20 ug total protein loaded in each lane. Values above each lane represent time post-induction.

## Slide 3
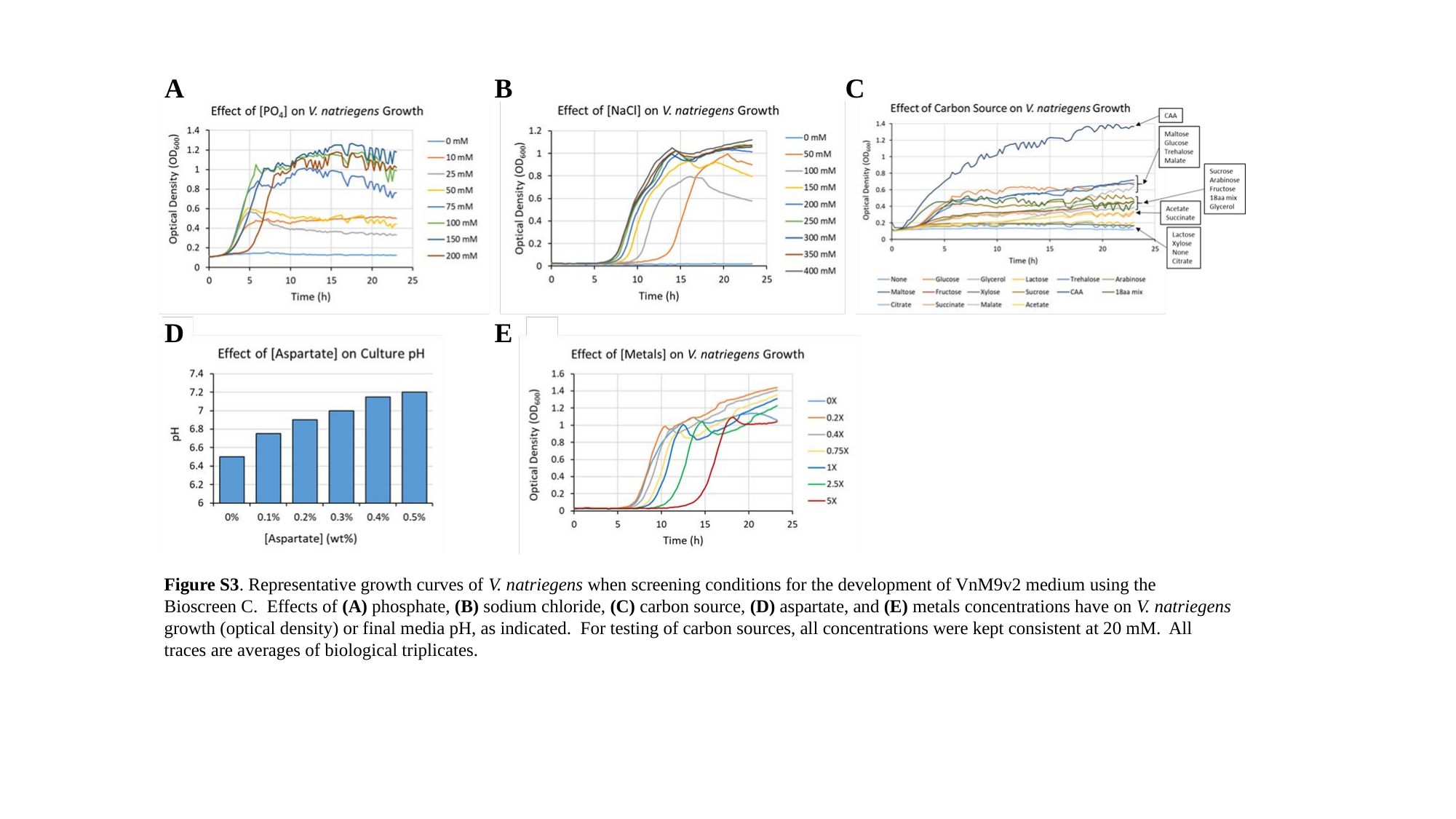

A
B
C
D
E
Figure S3. Representative growth curves of V. natriegens when screening conditions for the development of VnM9v2 medium using the Bioscreen C. Effects of (A) phosphate, (B) sodium chloride, (C) carbon source, (D) aspartate, and (E) metals concentrations have on V. natriegens growth (optical density) or final media pH, as indicated. For testing of carbon sources, all concentrations were kept consistent at 20 mM. All traces are averages of biological triplicates.

## Slide 4
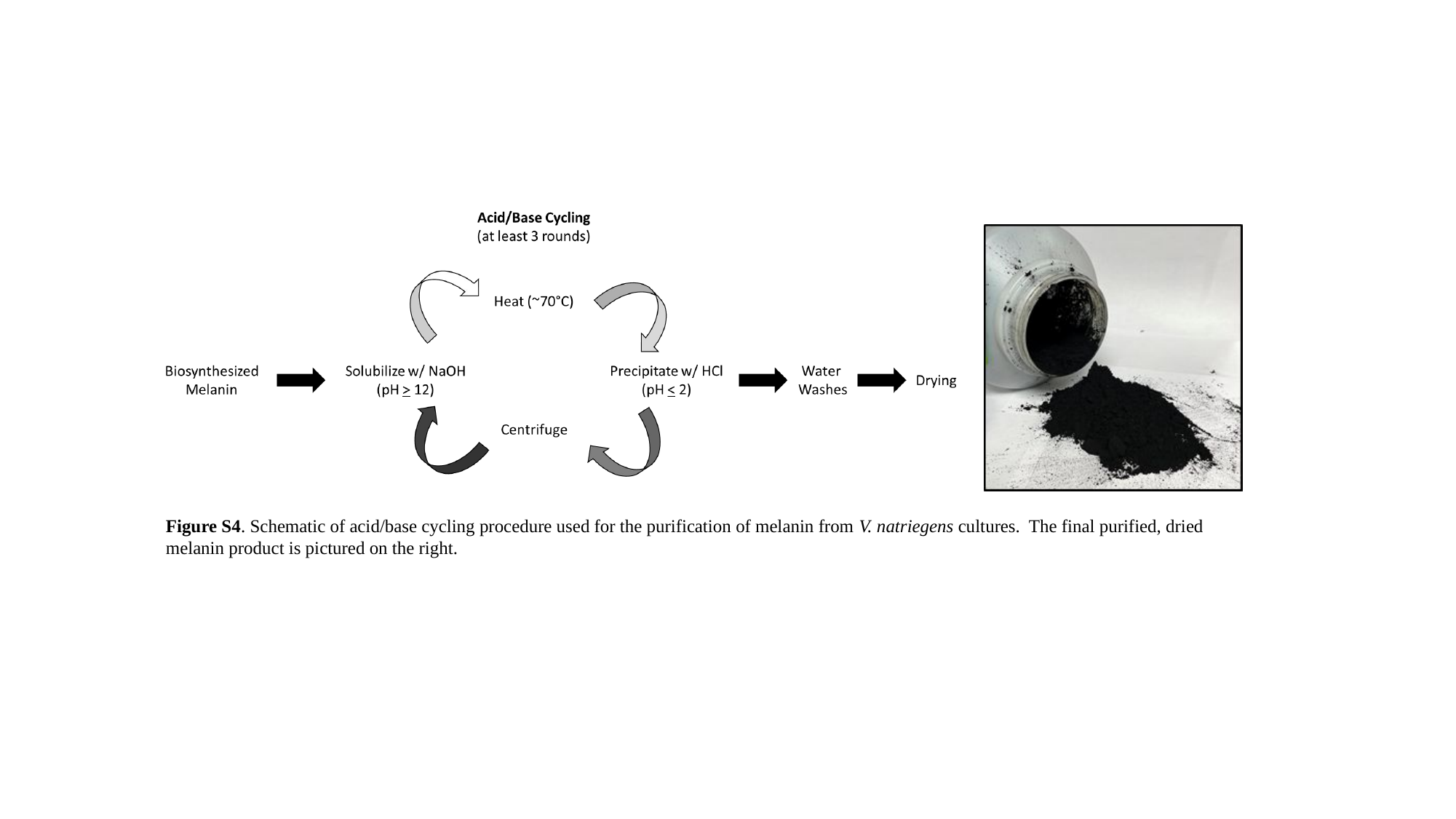

Figure S4. Schematic of acid/base cycling procedure used for the purification of melanin from V. natriegens cultures. The final purified, dried melanin product is pictured on the right.

## Slide 5
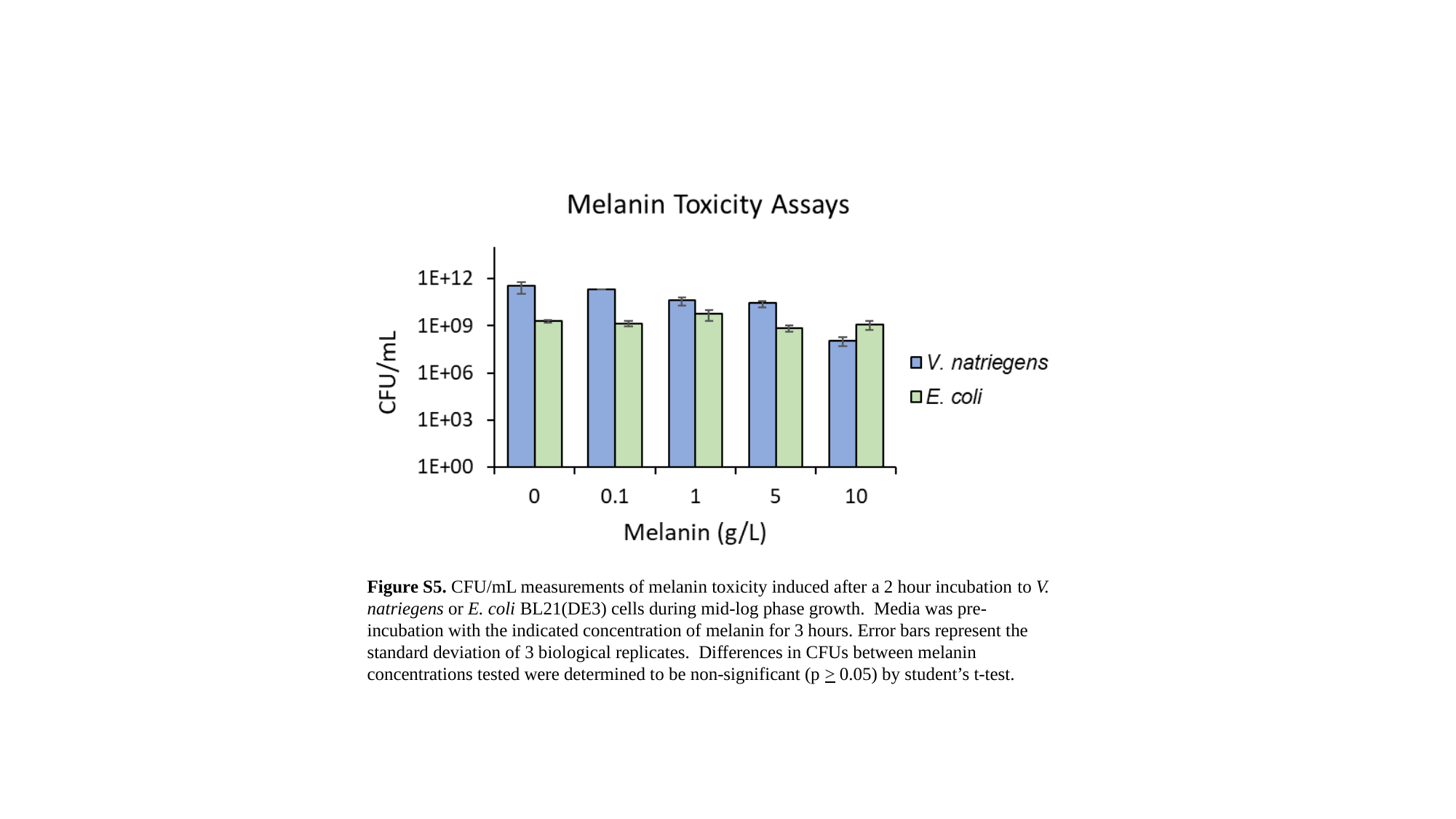

Figure S5. CFU/mL measurements of melanin toxicity induced after a 2 hour incubation to V. natriegens or E. coli BL21(DE3) cells during mid-log phase growth. Media was pre-incubation with the indicated concentration of melanin for 3 hours. Error bars represent the standard deviation of 3 biological replicates. Differences in CFUs between melanin concentrations tested were determined to be non-significant (p > 0.05) by student’s t-test.

## Slide 6
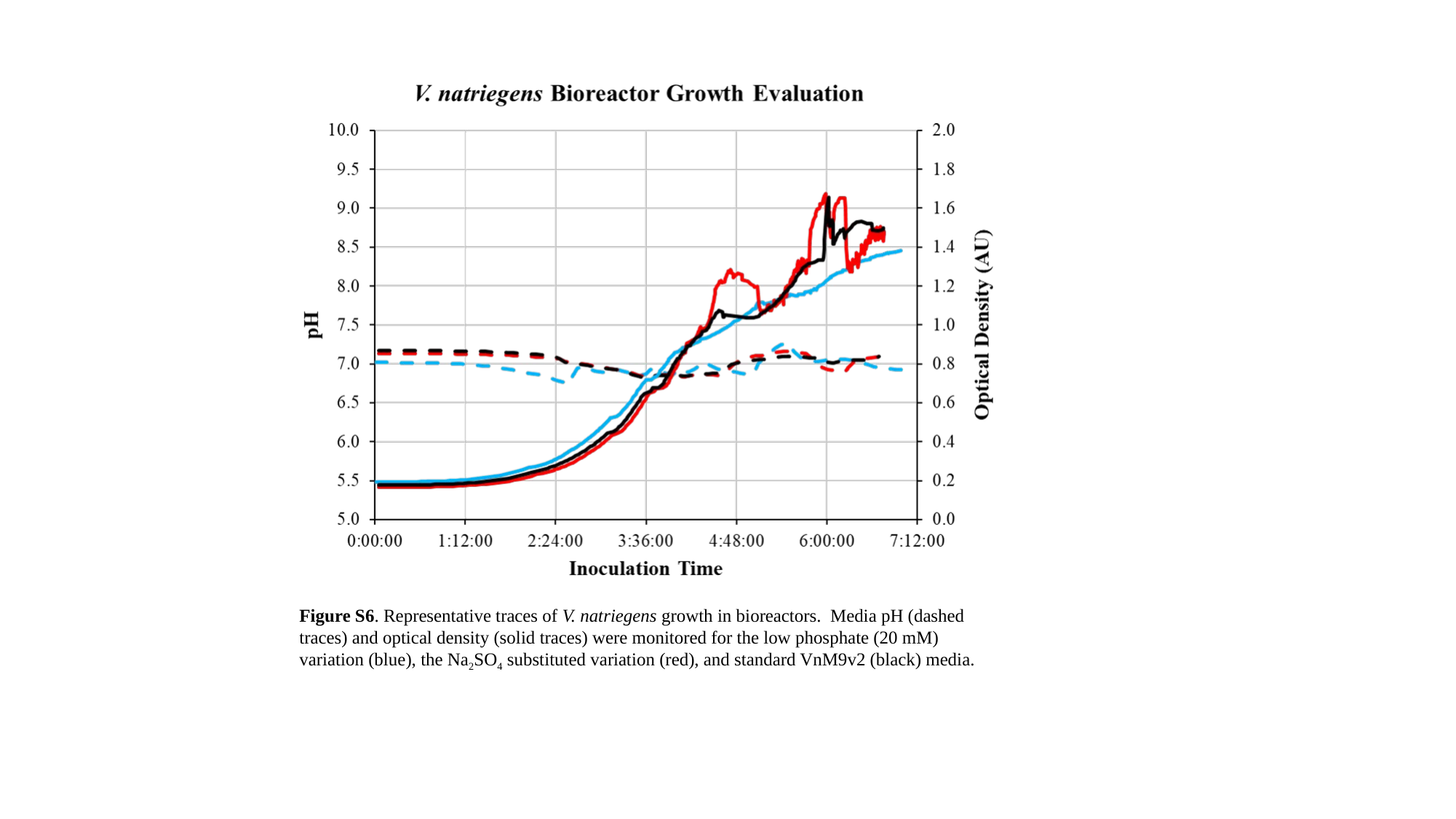

Figure S6. Representative traces of V. natriegens growth in bioreactors. Media pH (dashed traces) and optical density (solid traces) were monitored for the low phosphate (20 mM) variation (blue), the Na2SO4 substituted variation (red), and standard VnM9v2 (black) media.

## Slide 7
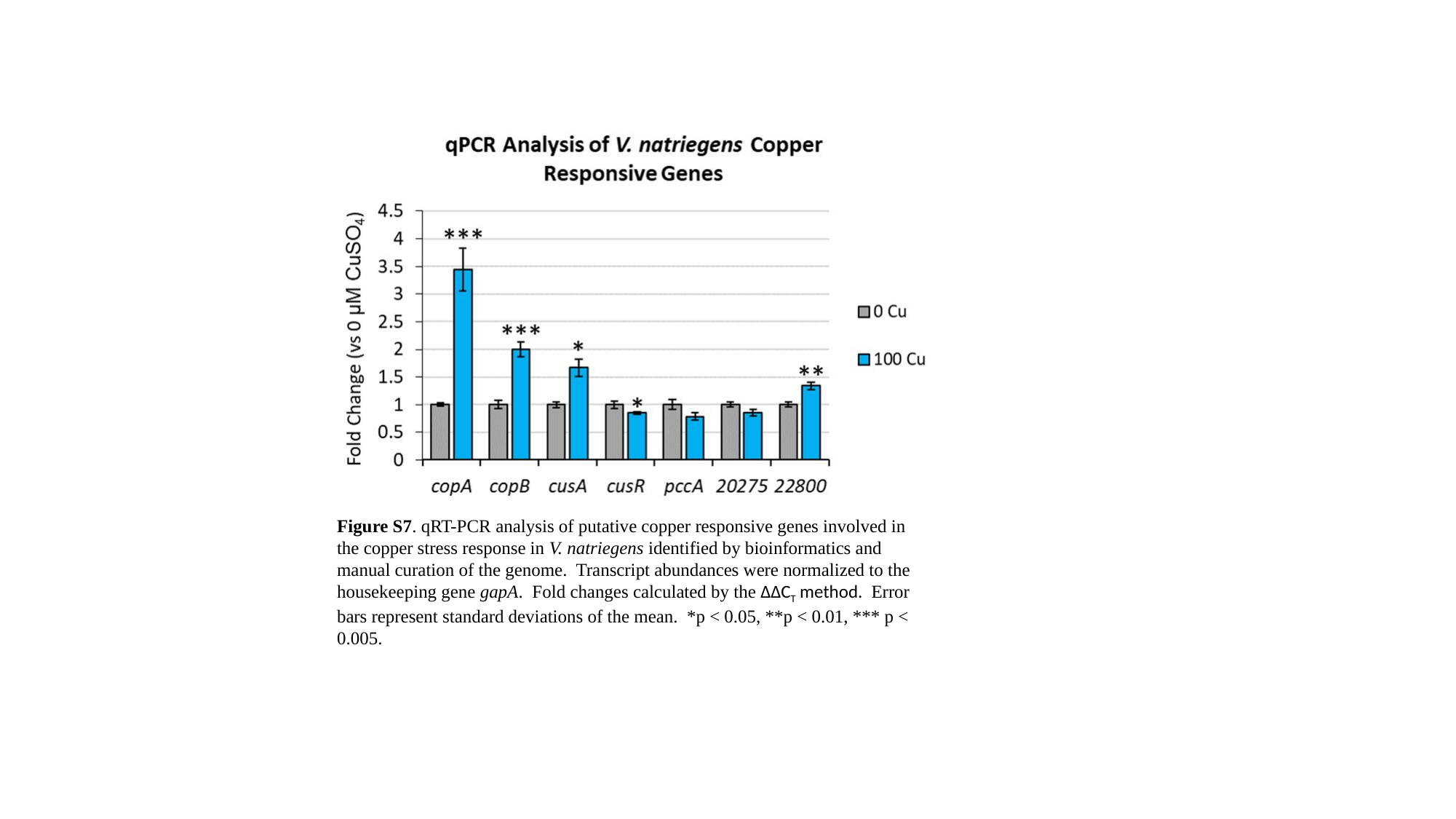

Figure S7. qRT-PCR analysis of putative copper responsive genes involved in the copper stress response in V. natriegens identified by bioinformatics and manual curation of the genome. Transcript abundances were normalized to the housekeeping gene gapA. Fold changes calculated by the ΔΔCT method. Error bars represent standard deviations of the mean. *p < 0.05, **p < 0.01, *** p < 0.005.
